# (±)-Polysiphenol and Other Analogues via Symmetrical Intermolecular Dimerizations: A Synthetic, Spectroscopic, Structural, and Computational Study

**DOI:** 10.1021/acs.jnatprod.2c00749

**Published:** 2022-10-26

**Authors:** D. Christopher Braddock, Anna Duran-Corbera, Masih Nilforoushan, Ziye Yang, Tianyou He, Gajan Santhakumar, Karim A. Bahou, Henry S. Rzepa, Rudiger Woscholski, Andrew J. P. White

**Affiliations:** Department of Chemistry, Molecular Sciences Research Hub, Imperial College London, White City Campus, 82 Wood Lane, London W12 0BZ, U.K.

## Abstract

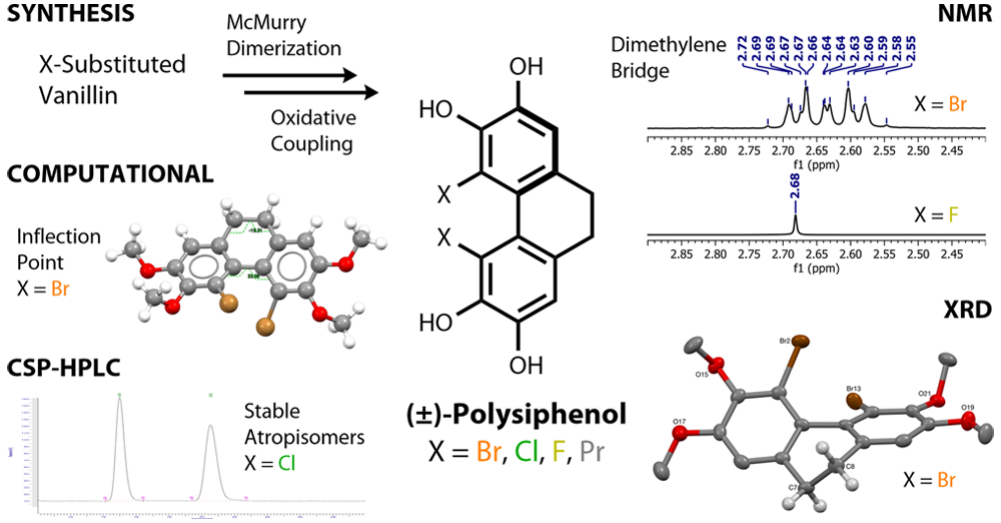

We report an improved total synthesis of 4,5-dibromo-9,10-dihydrophenanthrene-2,3,6,7-tetraol, (±)-polysiphenol, via intermolecular McMurray dimerization of 5-bromovanillin and subsequent intramolecular oxidative coupling as the key steps. The synthetic route is applicable to 4,5-dichloro- and 4,5-difluoro-halologues (as well as a 4,5-dialkyl-analogue). Distinctive AA′BB′ multiplets in their ^1^H NMR spectra for the dimethylene bridges of the dibromo and dichloro compounds reveal them to be room-temperature stable atropisomers, while for the difluoro compound they present as a singlet. X-ray crystal structure determinations of their tetramethylated synthetic precursors show atropisomeric twist angles of 48°, 46°, and 32°, respectively, with the former representing the largest yet observed in any 4,5-disubstituted-9,10-dihydrophenanthrene. DFT computational studies reveal an unprecedented two-stage atropisomeric interconversion process involving time-independent asynchronous rotations of the dimethylene bridge and the biaryl axis for halologues containing chlorine or bromine, but a more synchronous rotation for the difluoro analogue.

Marine red algae provide structurally diverse halogenated metabolites as stimulating structures for target synthesis and as prospective leads for medicinally potent entities.^1^ In 2011 we reported the first total synthesis of (±)-polysiphenol (**1a**),^2^ a compound isolated from *Polysyphonia ferulacea*, as an atropisomerically stable 4,5-dibrominated 9,10-dihydrophenanthrene and the first naturally occurring 9,10-dihydrophenanthrene from a marine source.^3^ Our original synthesis featured a one-pot telescoped bromination–phosphonium salt formation–Wittig reaction to join two nonidentical aromatic fragments, followed by a highly regioselective, biomimetically inspired, intramolecular oxidative coupling (Figure 1a). To date, this represents the only total synthesis as yet reported for (±)-polysiphenol, or related brominated metabolites with a phenanthrene skeleton.^4^ While some potent biological activity has been reported for these latter compounds, any biological activity of polysiphenol (**1a**) remains to be established. However, attempts to revisit our synthesis to provide further quantities of polysiphenol for such purposes revealed that the one-pot telescoped bromination–phosphonium salt formation–Wittig reaction could be capricious, returning the desired product in wildly varying yield (8–79%). Herein, in an effort to overcome these difficulties, and to take synthetic advantage of the inherent symmetry present in the target molecule, we report the direct McMurray dimerization of 5-bromovanillin as a more efficient approach to (±)-polysiphenol **1a** (Figure 1b). We show that this synthetic route can be readily adapted to provide its chlorinated and fluorinated halologues as compounds for putative structure–activity relationship (SAR) studies. X-ray crystal structure determinations of their tetra-*O*-methylated precursors provide the first experimental information about the extent of their atropisomeric twist angles, and computational studies provide insight into their atropisomeric stability and interconversion pathways.

**Figure 1:**
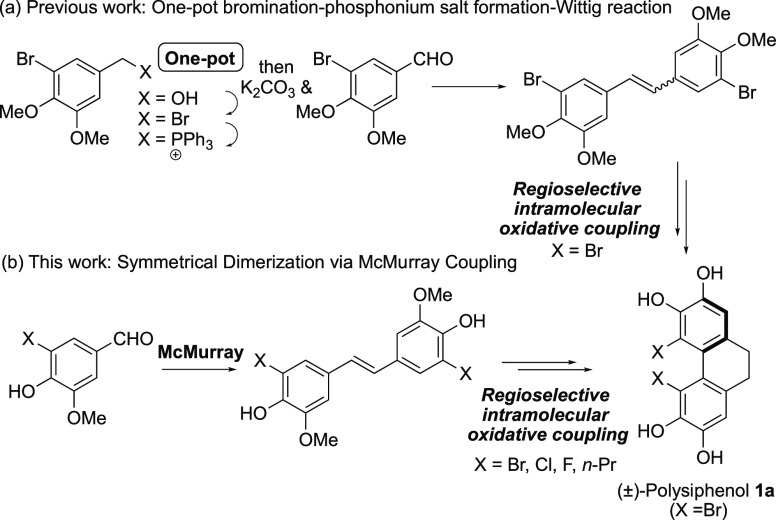
(a) Previous work. (b) This work.

## Results and Discussion

**Scheme 1:**
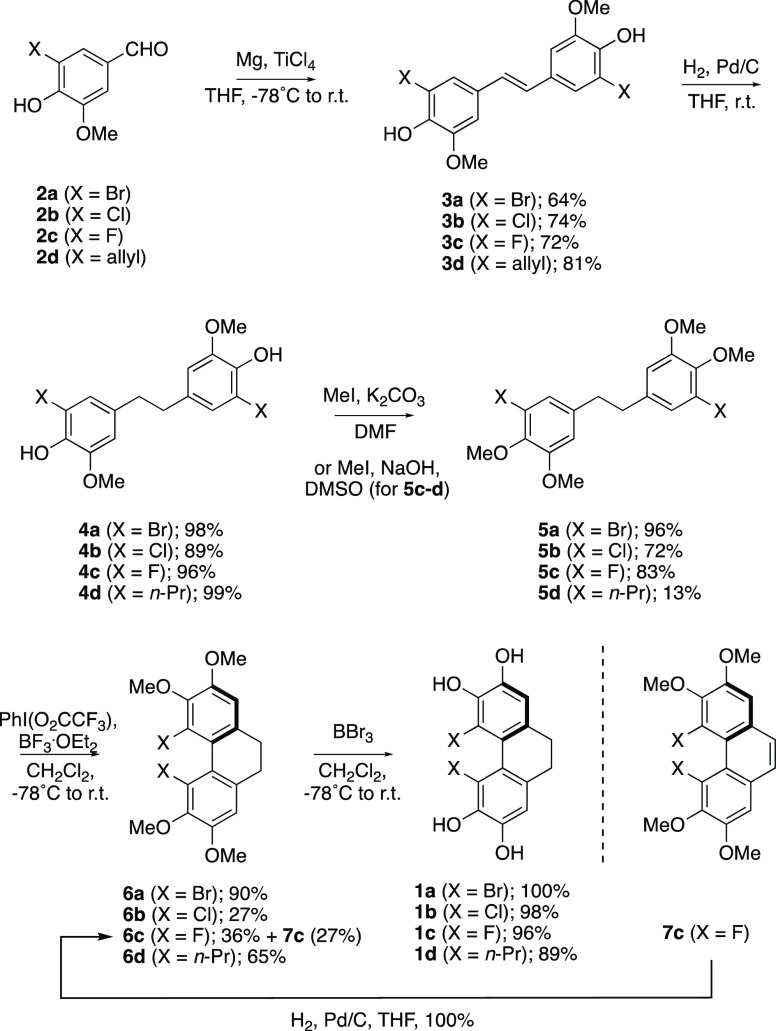
Synthesis of 4,5-Disubstituted-9,10-dihydrophenanthrene-2,3,6,7-tetraols **1a**–**1d**

In revisiting our synthetic approach to (±)-polysiphenol **1** we were aware that Harvey had recently reported the first direct McMurray dimerization of vanillin,^5^ by consideration and modification of previous work.^6^ Accordingly, we were drawn to investigate Harvey’s conditions for the direct McMurray dimerization of commercially available 5-bromovanillin **2a** (Scheme 1). Much to our delight, this procedure provided stilbene **3a**(7) in pure form directly after workup. Subsequent hydrogenation provided novel 4,4′-diphenol-1,2-ethane **4a**, which after dimethylation intersected with our previous route as intermediate **5a**.^2^ This three-step conversion of **2a** to **5a** requires no chromatographic purification and compares favorably to the previous four-step route by removing the capricious bromination–phosphonium salt formation–Wittig reaction step.^2^ Subsequent regioselective oxidative coupling to form the dibromodihydrophenanthrene **6a** and global demethylation both as previously reported^2^ provided (±)-polysiphenol **1a**.

The same synthetic sequence provided the 4,5-dichloro- and 4,5-difluoro-9,10-dihydrophenanthrene halologues **1b** and **1c** starting from commercially available 5-chloro- and 5-fluorovanillin **2b** and **2c** without significant complications (Scheme 1). In the case of the fluorine halologue, the oxidative coupling step (**5c** → **6c**) gave also overoxidized 4,5-difluorophenanthrene **7c**, which had not been observed in the oxidative couplings of dibromo (**5a** → **6a**) or dichloro (**5b** → **6b**) analogues.^8^ We attribute this different behavior to the ability of the smaller fluorine atoms to be more readily accommodated within the planar structure of a phenanthrene.^9^ This observation was also the first indication of the lack of atropisomeric stability in difluoride **6c** (and **1c**) (vide infra). It was found that difluorophenanthrene **7c** could be smoothly reduced back to dihydrophenanthrene **6c** using Pd/C and hydrogen gas in quantitative yield.

4,5-Dialkyl analogue **1d** was also produced starting from known 5-allyl vanillin **2d** (Scheme 1), the latter being readily obtained via aromatic Claisen rearrangement of *O*-allyl vanillin.^10^ This sequence also proceeded without complication, where the unsaturated allyl groups are transformed into *n*-propyl groups in the hydrogenation step (**3d** → **4d**), albeit with a subsequent difficult *O*-alkylation step (**4d** → **5d**), which did not improve regardless of the conditions employed (which we attribute to the amphiphilic nature of the substrate).

**Figure 2:**
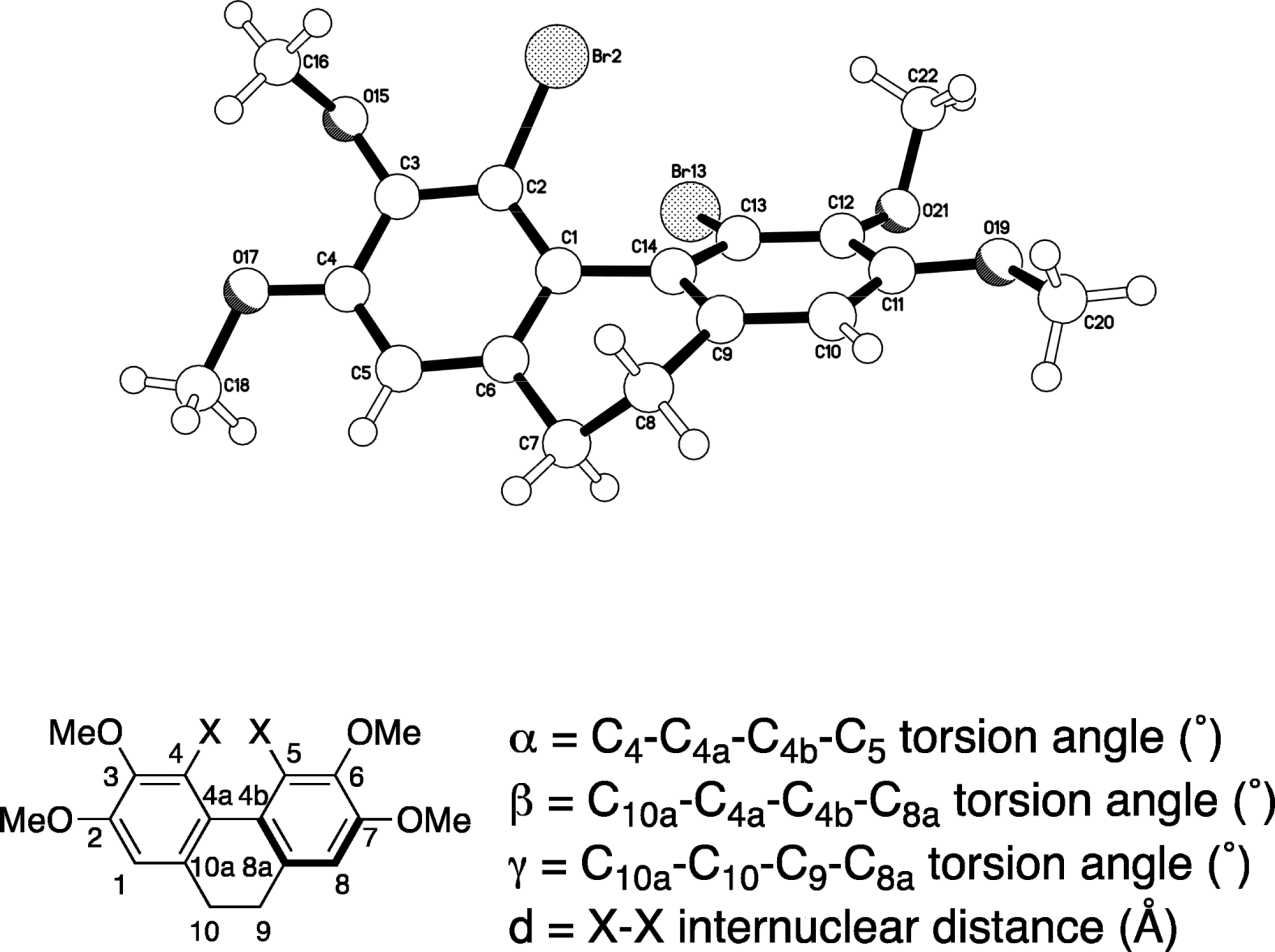
Top: X-ray crystal structure of dibromide **6a**. Bottom: Definition of the structural parameters for 4,5-dihalo/dialkyl-2,3,6,7-tetramethoxy-9,10-dihydrophenanthrenes (**6a**–**6d**).

As previously reported, the atropisomeric axis in polysiphenol **1a** gives rise to a distinctive AA′BB′ multiplet for the dimethylene bridge protons in its ^1^H NMR spectrum at room temperature.^3^ A similar pattern is also observed for dichloride **1b** and dipropyl **1d** prepared here, indicative of stable atropisomers also. In contrast, the methylene bridge for the difluoride **1c** presents as a singlet, indicating that the smaller fluorine atoms do not result in restricted rotation at this temperature.^11^ The ^1^H NMR spectra of tetramethyl precursors **6a**–**6d** show the same features, respectively. Pleasingly, we were able to obtain X-ray crystal structures for all four of these compounds for comparison (Figure 2 and Supporting Information).

Inspection of the crystal structure for 4,5-dibromo-9,10-dihydrophenanthrene **6a** (Figure 2, top) reveals an atropisomeric *C*_2_-symmetic twisted tricyclic core, as to be expected based on the above ^1^H NMR analysis and previous molecular mechanics modeling for polysiphenol.^3,12^ This is the first crystal structure of any 4,5-dibromo-9,10-dihydrophenanthrene,^13^ revealing an internuclear Br–Br distance of *d* = 3.43 Å, torsion angles for α = 47.8° and β = 37.3°, and a dimethylene bridge with minimal torsional strain (γ = 60.1°). The α-torsion twist angle is the largest yet to be observed in any 4,5-disubstituted-9,10-dihydrophenanthrene. Dipropyl derivative **6d** was found to have a smaller internuclear (benzylic methylene) C–C distance of 3.21 Å, but the torsion angles of 46.4° (α), 36.1° (β), and 61.0° (γ) reveal the dihydrophenanthrene unit to be essentially isostructural with dibromide **6a**.

For dichloride **6b** and difluoride **6c**, unsurprisingly, the internuclear distances between the two halogens decrease with the decreasing size of the halogen (**6b**, Cl–Cl, *d* = 3.17 Å; **6c**, F–F, *d* = 2.48 Å), and the observed α and β torsion angles decrease also (**6b**, α = 45.9°, β = 35.6°; **6c**, α = 31.5°, β = 25.4°), in line with previous observations.^14^ In both cases the dimethylene bridges retain essentially perfect γ torsion angles (**6b**, 62.1°; **6c**, 58.8°), presumably to minimize torsional strain, regardless of the variation in the other torsion angles. While the trend in X-ray structural parameters in the series **6a** to **6b** to **6c** is clearly consistent with the ^1^H NMR data, which suggest a switch over from atropisomerically stable (**6a**, **6b**)^15^ to non-atropisomerically stable (**6c**) structures at room temperature, we elected to undertake a computational study to assess the relative atropisomeric stability of 4,5-dihalo-9,10-dihydrophenanthrenes **6a**–**6c**.

**Figure 3:**
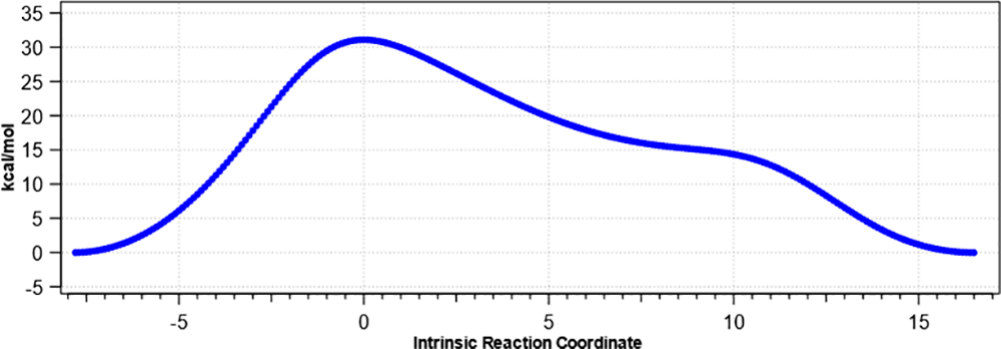
Computed relative energies as a function of intrinsic reaction coordinate for atropisomerism of dibromide **6a**, with diaryl rotations followed by methylene rotations.

A ωB97XD/6-31G(d,p) density functional procedure^16^ with a CHCl_3_ solvent field applied^17^ was deemed sufficiently accurate to explore the atropisomeric potential energy surfaces for **1**. The following combinations of halogens were used: Br, Br (**1a**), Cl,Br, Cl,Cl (**1b**), F,Cl, F,F (**1c**). Approximate transition states were initially located using relaxed scans and optimized accurately, and an intrinsic reaction coordinate (IRC)^18^ was obtained. That for dibromide **1a** is shown in Figure 3 and reveals a two-stage, albeit concerted, process in which the transition state (IRC = 0.0) involves rotation of predominantly the biaryl C–C bond—whereby the two halogens pass each other—which is then followed (IRC = 8) by rotation of the methylene–methylene bond—overall transforming γ from ca. 60° to ca. −60°—with a lower effective barrier (∼15 kcal/mol) (see ref (20) for links to animations). The calculated atropisomeric barriers reduce in height as the halogens become smaller, Δ*G*^⧧^ 30.5 (Br,Br, **1a**), 27.9 [23.4] (Me,Me), 27.5 (Br,Cl), 25.3 [22.6] (Cl,Cl, **1b**), 14.7 (Cl,F), and 10.5 [11.1] (F,F **1c**) kcal/mol, and show reasonable agreement with experimentally measured values^19^ (in square brackets). At ambient temperatures therefore, the combinations involving bromine, chlorine, and methyl will show no NMR exchange effects due to atropisomerism, while the low F,F barrier would result in fully averaged NMR behavior, as seen experimentally. The combination Cl,F is predicted to show intermediate NMR behavior.

**Figure 4:**
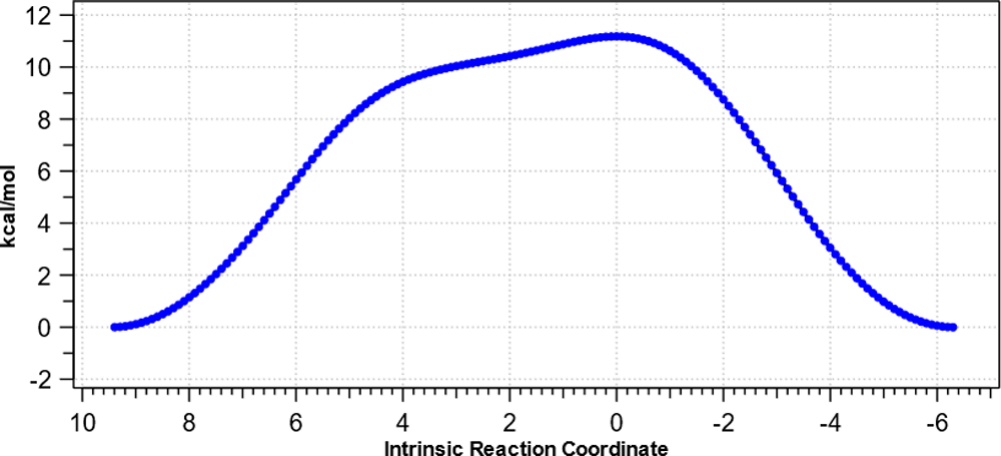
Computed relative energies as a function of intrinsic reaction coordinate for atropisomerism of difluoride **6c**, with dimethylene rotations followed by diaryl rotations.

As the halogen size decreases, the nature of the two-stage atropisomeric process evolves from one in which the transition state corresponds to predominant halogen atropisomerism at the diaryl bond to one where it more closely resembles methylene rotation, the latter achieved only when both halogens are fluorine (Figure 4).

In conclusion, we have developed a total synthesis of (±)-polysiphenol **1a** and its chlorinated and fluorinated halologues, **1b** and **1c**, respectively, as well as dialkyl analogue **1d** via intermolecular McMurray dimerization and subsequent intramolecular oxidative coupling as the key steps. A combined spectroscopic, crystallographic, and computational study has shown that the dibromide **1a**, dichloride **1b**, and dialkyl **1d** derivatives are atropisomerically stable at room temperature, but where the difluoride **1c** is not^20^ and where the mixed fluoro/chloro halide is predicted to show intermediate behavior.

## Experimental Section

### General Experimental Procedures

See Supporting Information.

### (*E*)-4,4′-(Ethene-1,2-diyl)bis(2-bromo-6-methoxyphenol) (**3a**)

TiCl_4_ (1.89 mL, 17.2 mmol, 2.0 equiv) was added dropwise to a stirred suspension of Mg (420 mg, 17.2 mmol, 2.0 equiv) in THF (100 mL) at −78 °C. The mixture was allowed to warm to rt and became black. After stirring for 1 h, a solution of bromovanillin **2a** (2.00 g, 8.6 mmol) in THF (20 mL) was added dropwise and was stirred overnight. The solvent was removed in vacuo, and the black solid mass was treated with 2 M aqueous HCl (200 mL). The resulting suspended solids were collected by vacuum filtration, washed successively with H_2_O and *n*-hexane, and dried under vacuum to yield dibromostilbene **3a** (1.20 g, 2.79 mmol, 65%) as an off-white solid: IR (neat) 3433 (br) cm^–1^; ^1^H NMR (400 MHz; DMSO-*d*_6_) δ 9.55 (s, 2H), 7.27 (d, *J* = 1.8 Hz, 2H), 7.19 (d, *J* = 1.8 Hz, 2H), 7.04 (s, 2H), 3.88 (s, 6H) ppm; ^13^C NMR (100 MHz; DMSO-*d*_6_) δ 148.6, 143.3, 129.8, 125.9, 122.7, 109.6, 108.5, 56.2 ppm; HRMS (ES^–^, TOF) *m*/*z*: 426.9196 [M – H]^−^ (calcd for C_16_H_13_^79^Br_2_O_4_, 426.9181).

### 4,4′-(Ethane-1,2-diyl)bis(2-bromo-6-methoxyphenol) (**4a**)

Pd/C (10 wt %, 320 mg) was added to a solution of stilbene **3a** (1.20 g, 2.79 mmol) in THF (80 mL) at rt, and the resulting suspension was stirred vigorously under an atmosphere of hydrogen gas. After 24 h, the mixture was filtered and evaporated to give dibromoethane **4a** (1.20 g, 2.54 mmol, 98%) as a pale pink solid: IR (neat) 3376 (br), 2956, 2927, 2864 cm^–1^; ^1^H NMR (400 MHz, DMSO-*d*_6_) δ 9.14 (s, 2H), 6.94 (d, *J* = 1.8 Hz, 2H), 6.83 (d, *J* = 1.8 Hz, 2H), 3.78 (s, 6H), 2.72 (s, 4H) ppm; ^13^C NMR (100 MHz, DMSO-*d*_6_) δ 148.2, 141.7, 133.6, 123.8, 111.6, 109.0, 56.1, 36.5 ppm.

### 1,2-Bis(3-bromo-4,5-dimethoxyphenyl)ethane (**5a**)

To a stirred solution of bis(methoxyphenol) **4a** (1.0 g, 2.3 mmol) in dimethylformamide (DMF) (25 mL) at rt was added potassium carbonate (950 mg, 6.9 mmol, 3.0 equiv). After 10 min, methyl iodide (0.29 mL, 4.6 mmol, 2.0 equiv) was added, the mixture was stirred overnight at 50 °C, the solvent was evaporated, and H_2_O was added. The aqueous phase was extracted with diethyl ether (3 × 200 mL), and the combined organics were washed with saturated aqueous sodium carbonate solution (200 mL) and brine (200 mL), dried over sodium sulfate, and evaporated to yield the dibromotetramethyl ether **5a** (1.02 g, 96%) as a white crystalline solid: mp 105–108 °C (lit.^2^ 102 °C); IR (neat) 3002, 2965, 2933 cm^–1^; ^1^H NMR (400 MHz, CDCl_3_) δ 6.96 (d, *J* = 1.8 Hz, 2H), 6.57 (d, *J* = 1.8 Hz), 3.83 (s, 6H), 3.81 (s, 6H), 2.80 (s, 4H) ppm; ^13^C NMR (100 MHz, CDCl_3_) δ 153.6, 144.9, 138.5, 124.5, 117.5, 112.3, 60.7, 56.2, 37.5 ppm.

### (*E*)-4,4′-(Ethene-1,2-diyl)bis(2-chloro-6-methoxyphenol) (**3b**)

According to the procedure for stilbene **3a**, using 5-chlorovanillin **2b** (1.00 g, 5.35 mmol, 1.0 equiv), Mg (261 mg, 10.7 mmol, 2.0 equiv), and TiCl_4_ (1.18 mL, 10.7 mmol, 2.0 equiv) in THF gave dichlorostilbene **3b** (677 mg, 1.98 mmol, 74%) as a pink crystalline solid: mp 231–237 °C; IR (neat) 3467 (br) cm^–1^; ^1^H NMR (400 MHz; DMSO-*d*_6_) δ 9.50 (s, 2H), 7.16–7.14 (m, 4H), 7.06 (s, 2H), 3.88 (s, 6H) ppm; ^13^C NMR (100 MHz; DMSO-*d*_6_) δ 148.9, 142.2, 129.1, 126.1, 120.4, 119.8, 108.0, 56.2 ppm; HRMS (APCI, Orbitrap) *m*/*z* 339.0183 [M – H]^−^ (calcd for C_16_H_13_^35^Cl_2_O_4_, 339.0185).

### 4,4′-(Ethane-1,2-diyl)bis(2-chloro-6-methoxyphenol) (**4b**)

According to the procedure for diarylethane **4a**, using stilbene **3b** (500 mg, 1.47 mmol, 1.0 equiv) and Pd/C (10 wt %, 344 mg) in THF (45 mL) gave dichloroethane **4b** (448 mg, 1.31 mmol, 89%) as a dark gray solid: IR (neat) 3396 (br), 2929 cm^–1^; ^1^H NMR (400 MHz, DMSO-*d*_6_) δ 9.10 (s, 2H), 6.79 (s, 4H), 3.78 (s, 6H), 2.73 (s, 4H) ppm; ^13^C NMR (100 MHz, DMSO-*d*_6_) δ 148.5, 140.7, 132.8, 121.0, 119.4, 111.1, 56.1, 36.5 ppm; HRMS (ES^–^, TOF) *m*/*z* 341.0353 [M – H]^−^ (calcd for C_16_H_15_^35^Cl_2_O_4_, 341.0347).

### 1,2-Bis(3-chloro-4,5-dimethoxyphenyl)ethane (**5b**)

According to the procedure for tetramethyl ether **5a**, using bis(methoxyphenol) **4b** (385 mg, 1.12 mmol, 1.0 equiv), potassium carbonate (645 mg, 4.77 mmol, 4.3 equiv), and methyl iodide (0.70 mL, 12.5 mmol, 11.2 equiv) in DMF (13 mL) for 5 days at rt gave dichlorotetramethyl ether **5b** (301 mg, 0.81 mmol, 72%) as a yellow solid: IR (neat) 2968, 2928, 2860, 2830, cm^–1^; ^1^H NMR (400 MHz, CDCl_3_) δ 6.80 (d, *J* = 2.2 Hz, 2H), 6.54 (d, *J* = 2.2 Hz, 2H), 3.84 (s, 6H), 3.82 (s, 6H), 2.81 (s, 4H) ppm; ^13^C NMR (100 MHz, CDCl_3_) δ (ppm) 153.7, 143.9, 137.9, 128.2, 121.8, 111.6, 60.8, 56.2, 37.5 ppm; HRMS (ES^+^, TOF) *m*/*z*: 403.1087 [M + MeOH + H]^+^ (calcd for C_19_H_25_^35^Cl_2_O_5_, 403.1079).

### 4,5-Dichloro-2,3,6,7-tetramethoxy-9,10-dihydrophenanthrene (**6b**)

To a stirred solution of diarylethane **5b** (160 mg, 0.43 mmol, 1.0 equiv) in CH_2_Cl_2_ (12 mL) at −78 °C were added [bis(trifluoroacetoxy)iodo]benzene (224 mg, 0.52 mmol, 1.2 equiv) and BF_3_·OEt_2_ (0.14 mL, 1.10 mmol, 2.5 equiv). The solution was allowed to warm to rt and stirred for 28 h. The mixture was filtered through a silica plug, and the volatiles were evaporated and recrystallization first from EtOAc and *n*-hexane and then from CH_2_Cl_2_ to give dichlorodihydrophenanthrene **6b** (44 mg, 0.1 mmol, 28%) as colorless blocky needles: mp 161–165 °C; IR (neat) 3001, 2941, 2836 cm^–1^; ^1^H NMR (400 MHz, CDCl_3_) δ 6.78 (s, 2H), 3.91 (s, 6H), 3.90 (s, 6H), 2.72–2.54 (m, 4H) ppm; ^13^C NMR (100 MHz, CDCl_3_) δ 152.4, 144.9, 137.5, 128.2, 125.8, 109.8, 60.9, 56.3, 31.5 ppm; HRMS (ES^+^, TOF) *m*/*z* 333.0906 [M – Cl]^+^ (calcd for C_18_H_18_^35^ClO_4_, 333.0894).

### 4,5-Dichloro-9,10-dihydrophenanthrene-2,3,6,7-tetraol (**1b**)

A solution of BBr_3_ in CH_2_Cl_2_ (1M, 0.37 mL, 0.37 mmol, 5.8 equiv) was added dropwise to tetramethyl ether **6b** (24 mg, 0.06 mmol) in CH_2_Cl_2_ (1.7 mL) at −78 °C. The reaction mixture was allowed to warm to rt and stirred for 24 h, and the volatiles were evaporated to afford dichlorotetrol **1b** (20 mg, 0.06 mmol, 98%) as a black solid: IR (neat) 3286 (br), 2939, 2855 cm^–1^; ^1^H NMR (400 MHz, CD_3_OD) δ 8.46 (s, 4H), 6.67 (s, 2H), 2.58–2.37 (m, 4H) ppm; ^13^C NMR (100 MHz, DMSO-*d*_6_) δ 144.8, 140.7, 132.3, 123.7 120.1, 112.6, 30.5 ppm; HRMS (ES^–^, TOF) *m*/*z* 310.9883 [M – H]^−^ (calcd for C_14_H_9_^35^Cl_2_O_4_, 310.9878).

### (*E*)-4,4′-(Ethene-1,2-diyl)bis(2-fluoro-6-methoxyphenol) (**3c**)

According to the procedure for stilbene **3a**, using 5-fluorovanillin **2c** (0.20 g, 1.17 mmol, 1.0 equiv), Mg (60 mg, 2.47 mmol), and TiCl_4_ (0.32 mL, 2.94 mmol) in THF gave difluorostilbene **3c** (129 mg, 0.42 mmol, 72%) as an orange solid: IR (neat) 3460 (br) cm^–1^; ^1^H NMR (400 MHz, DMSO-*d*_6_) δ 9.31 (s, 2H), 7.03–6.97 (m, 6H), 3.86 (s, 6H) ppm; ^13^C NMR (100 MHz, DMSO-*d*_6_) δ 151.6 (d, *J* = 236 Hz), 149.6 (d, *J* = 7 Hz), 133.9 (d, *J* = 15 Hz), 128.1 (d, *J* = 9 Hz), 126.5, 106.4 (d, *J* = 20 Hz), 105.7, 56.1 ppm; ^19^F{^1^H} NMR (376 MHz, DMSO-*d*_6_) −135.8 ppm; HRMS (ES^–^, TOF) *m*/*z* 307.0777 [M – H]^−^ (calcd for C_16_H_13_F_2_O_4_, 307.0782).

### 4,4′-(Ethane-1,2-diyl)bis(2-fluoro-6-methoxyphenol) (**4c**)

According to the procedure for diarylethane **4a**, using stilbene **3c** (188 mg, 0.61 mmol, 1.0 equiv) and Pd/C (10 wt %, 163 mg) in THF (17.5 mL) gave difluoroethane **4c** (148 mg, 0.48 mmol, 78%) as a white solid: IR (neat) 3332 (br) cm^–1^; ^1^H NMR (400 MHz, DMSO-*d*_6_) δ 8.88 (s, 2H), 6.65–6.61 (m, 4H), 3.76 (s, 6H) 2.73 (s, 4H) ppm; ^13^C NMR (100 MHz, DMSO-*d*_6_) δ 151.2 (d, *J* = 235 Hz), 149.3 (d, *J* = 9 Hz), 132.1 (d, *J* = 14 Hz), 132.0, 108.1, 108.0, 56.1, 36.5 ppm; ^19^F{^1^H} NMR (376 MHz, DMSO-*d*_6_) −136.2 ppm; HRMS (ES^–^, TOF) *m*/*z*: 309.0925 [M – H]^−^ (calcd for C_16_H_15_F_2_O_4_, 309.0938).

### 1,2-Bis(3-fluoro-4,5-dimethoxyphenyl)ethane (**5c**)

Methyl iodide (0.13 mL, 2.1 mmol, 10 equiv) was added to a stirred solution of potassium hydroxide (0.047 g, 0.84 mmol, 4 equiv) and bis(methoxyphenol) **4c** (65 mg, 0.21 mmol) in DMSO (2 mL) and stirred overnight at rt. Water was added, the mixture was extracted with diethyl ether (4 × 10 mL), and the combined organics were washed successively with H_2_O (3 × 10 mL), saturated aqueous sodium carbonate solution (2 × 10 mL), and brine (10 mL). The resulting organic layer was dried over magnesium sulfate, evaporated, and chromatographed (EtOAc:PE 2:3) to give difluorotetramethyl ether **5c** (59 mg, 0.17 mmol, 83%) as a white solid: IR 2930, 2836 cm^–1^; ^1^H NMR (400 MHz, CDCl_3_) δ 6.57 (dd, *J* = 11.2, 1.9 Hz, 2H), 6.43 (t, *J* = 1.6 Hz, 2H), 3.90 (s, 6H), 3.82 (s, 6H, OMe), 2.81 (s, 4H) ppm; ^13^C NMR (100 MHz, CDCl_3_) δ 156.0 (d, *J* = 244 Hz), 153.5 (d, *J* = 6 Hz), 137.0 (d, *J* = 8 Hz), 135.4 (d, *J* = 13 Hz), 108.9 (d, *J* = 19 Hz), 108.2, 61.7, 56.4, 37.6 ppm; ^19^F{^1^H} NMR (376 MHz, CDCl_3_) δ (ppm) −131.2; HRMS (CI^+^, Orbitrap) *m*/*z* 339.1402 [M + H]^+^ (calcd for C_18_H_21_F_2_O_4_, 339.1402).

### 4,5-Difluoro-2,3,6,7-tetramethoxy-9,10-dihydrophenanthrene (**6c**) and 4,5-difluoro-2,3,6,7-tetramethoxyphenanthrene (**7c**)

According to the procedure for dihydrophenathrene **6b**, using diarylethane **5c** (45 mg, 0.13 mmol), with [bis(trifluoroacetoxy)iodo]benzene (858 mg, 0.20 mmol, 1.5 equiv) and BF_3_·OEt_2_ (0.05 mL, 0.4 mmol, 3 equiv) in CH_2_Cl_2_ (12 mL), followed by chromatography (CH_2_Cl_2_:PE 2:1) gave first difluorophenanthrene **7c** (0.012 g, 0.036 mmol, 27%) as a white solid: *R*_*f*_ 0.38 (CH_2_Cl_2_:PE 2:1); ^1^H NMR (400 MHz, CDCl_3_) δ 7.50 (s, 2H), 7.05 (s, 2H), 4.07 (s, 6H), 4.02 (s, 6H) ppm; ^19^F{^1^H} NMR (376 MHz, CDCl_3_) δ −120.7 ppm; HRMS (CI^+^, Orbitrap) *m*/*z* 335.1077 [M + H]^+^ (calcd for C_18_H_17_F_2_O_4_, 335.1089), and second difluorodihydrophenanthrene **6c** (16 mg, 0.048 mmol, 36%) as a white solid: *R*_*f*_ 0.31 (CH_2_Cl_2_:PE 2:1); mp 172–174 °C; IR 2930 cm^–1^; ^1^H NMR (400 MHz, CDCl_3_) δ 6.63 (s, 2H), 3.94 (s, 6H), 3.90 (s, 6H), 2.68 (s, 4H) ppm; ^13^C NMR (100 MHz, CDCl_3_) δ 153.4 (AA′XX′ sextet, *J*_FF′_ = 138.3 Hz, *J*_FC_ = 188.2 Hz, *J*_FC′_ = 61.9 Hz), 152.4, 136.4 (t, *J* = 8 Hz), 134.4, 112.5 (t, *J* = 6 Hz), 106.9, 61.8, 56.4, 30.2 ppm; ^13^C{^19^F} NMR (100 MHz, CDCl_3_) δ 153.4, 152.4, 136.5, 134.5, 112.5, 106.9 (d, *J* = 158 Hz), 61.8 (q, *J* = 144 Hz), 56.4 (q, *J* = 144 Hz), 30.5 (t, *J* = 130 Hz); ^19^F{^1^H} NMR (376 MHz; CDCl_3_) δ (ppm) −125.9; HRMS (CI^+^, Orbitrap) *m*/*z* 337.1242 [M + H]^+^ (calcd for C_18_H_19_F_2_O_4_, 337.1246).

### 4,5-Difluoro-9,10-dihydrophenanthrene-2,3,6,7-tetraol (**1c**)

According to the procedure for tetrol **1b**, using tetramethyl ether **6c** (10 mg, 0.03 mmol) in CH_2_Cl_2_ (1 mL) and a solution of BBr_3_ in CH_2_Cl_2_ (1M, 0.2 mL, 0.2 mmol, 6 equiv) followed by quenching with MeOH and removal of the volatiles gave difluorotetrol **1c** (8 mg, 96%) as a brown solid: IR (neat,) 3291 (br), 2922, cm^–1^; ^1^H NMR (400 MHz, CD_3_OD) δ (ppm) 8.45 (s, 4H), 6.53 (s, 2H), 2.52 (s, 4H); ^19^F{^1^H} NMR (376 MHz, CD_3_OD) δ −133.8 ppm; HRMS (CI^–^, Orbitrap) *m*/*z* 279.0473 [M – H]^−^ (calcd for C_14_H_9_F_2_O_4_, 279.0463).

### (*E*)-4,4′-(Ethene-1,2-diyl)bis(2-allyl-6-methoxyphenol) (**3d**)

According to a modified procedure for stilbene **3a**, 5-allylvanillin **2d** (2.88 g, 15 mmol, 1.0 equiv), Mg (720 mg, 30 mmol, 2.0 equiv), and TiCl_4_ (3.3 mL, 30 mmol, 2 equiv) were refluxed in THF for 16 h. After evaporation of the solvent and treatment with 2 M aqueous HCl (300 mL), the mixture was extracted with EtOAc (4 × 50 mL), and the combined organics were washed successively with 2 M aqueous HCl (100 mL), distilled H_2_O (2 × 1 00 mL), and brine (2 × 100 mL), dried over MgSO_4_, and evaporated to give diallylstilbene **3d** (2.14 g, 6.1 mmol, 81%) as a red solid: IR (neat) 3410, 3075, 2933 cm^–1^; ^1^H NMR (400 MHz, CDCl_3_) δ 6.91 (s, 2H), 6.89 (s, 2H), 6.86 (s, 2H), 6.03 (ddt, *J* = 16.7, 10.0, 6.5 Hz, 2H), 5.71 (s, 2H), 5.16–5.04 (m, 4H), 3.94 (s, 6H), 3.42 (m, 4H) ppm; ^13^C NMR (100 MHz, CDCl_3_) δ 146.7, 143.2, 136.7, 129.6, 126.6, 125.9, 121.2, 115.8, 106.1, 56.2, 34.0 ppm; HRMS (CI^–^, Orbitrap) *m*/*z* 353.1739 [M + H]^+^ (calcd for C_22_H_25_O_4_, 353.1747).

### 4,4′-(Ethane-1,2-diyl)bis(2-methoxy-6-propylphenol) (**4d**)

According to the procedure for diarylethane **4a**, using stilbene **3d** (705 mg, 2.00 mmol) and Pd/C (10 wt %, 100 mg) in THF (40 mL) gave dipropylethane **4d** (707 mg, 1.98 mmol, 99%) as a wine red, sticky solid, which was used without further purification: IR (neat) 3540 (br), 2957, 2931, 2868, cm^–1^; ^1^H NMR (400 MHz, CDCl_3_) δ 6.58 (s, 2H), 6.51 (s, 2H), 5.52 (s, 2H), 3.84 (s, 6H), 2.78 (s, 4H), 2.58 (t, *J* = 7.6 Hz, 4H), 1.62 (sextet, *J* = 7.4 Hz, 4H), 0.96 (t, *J* = 7.4 Hz, 6H) ppm; ^13^C NMR (100 MHz, CDCl_3_) δ 146.1, 141.6, 132.9, 128.2, 122.3, 108.7, 56.1, 38.4, 32.0, 23.2, 14.2 ppm.

### 1,2-Bis(3,4-dimethoxy-5-propylphenyl)ethane (**5d**)

According to the procedure for tetramethyl ether **5c**, using bis(methoxyphenol) **4d** (600 mg, 1.67 mmol, 1 equiv), methyl iodide (1.1 mL, 17.7 mmol, 10.6 equiv), and potassium hydroxide (380 mg, 6.8 mmol, 4 equiv) in DMSO (50 mL), extracting with CH_2_Cl_2_, and purification by chromatography (EtOAc:PE 3:17) gave dipropyltetramethyl ether **5d** (86 mg, 0.22 mmol, 13%) as white crystals: mp 62–64 °C; IR (neat) 2957, 2934, 2868 cm^–1^; ^1^H NMR (400 MHz, CDCl_3_) δ 6.59 (s, 2H), 6.54 (s, 2H), 3.81 (s, 6H), 3.79 (s, 6H), 2.83 (s, 4H), 2.57 (t, *J* = 7.4 Hz, 4H), 1.59 (sextet, *J* = 7.4 Hz, 4H), 0.96 (t, *J* = 7.4 Hz, 6H) ppm; ^13^C NMR (100 MHz, CDCl_3_) δ 152.5, 145.5, 137.4, 136.2, 122.0, 110.6, 60.8, 55.8, 38.1, 32.0, 24.1, 14.3 ppm; HRMS (CI^+^, Orbitrap) *m*/*z* 387.2531 [M + H]^+^ (calcd for C_24_H_35_O_4_, 387.2530).

### 2,3,6,7-Tetramethoxy-4,5-dipropyl-9,10-dihydrophenanthrene (**6d**)

According to the procedure for dihydrophenanthrene **6b**, using diarylethane **5d** (80 mg, 0.21 mmol, 1 equiv) with [bis(trifluoroacetoxy)iodo]benzene (107 mg, 0.25 mmol, 1.2 equiv) and BF_3_·OEt_2_ (65 μL, 0.52 mmol, 2.5 equiv) in CH_2_Cl_2_ (10 mL), followed by chromatography (EtOAc:PE 3:17), gave dipropyldihydrophenanthrene **6d** (52 mg, 0.14 mmol, 65%) as ivory needles: mp 118–121 °C; IR (neat) 2958, 2928, 2869, 2836 cm^–1^; ^1^H NMR (400 MHz, CDCl_3_) δ 6.70 (s, 2H), 3.89 (s, 6H), 3.86 (s, 6H), 2.98–2.86 (m, 2H), 2.62–2.43 (m, 6H), 1.34 (m, 2H), 1.07 (m, 2H), 0.52 (t, *J* = 7.3 Hz, 6H); ^13^C NMR (100 MHz, CDCl_3_) δ 150.9, 146.5, 136.3, 134.3, 128.6, 108.9, 61.1, 55.8, 31.9, 31.3, 24.2, 13.9; HRMS (CI^+^, Orbitrap) *m*/*z* 385.2380 [M + H]^+^ (calcd for C_24_H_33_O_4_, 385.2373).

### 4,5-Dipropyl-9,10-dihydrophenanthrene-2,3,6,7-tetraol (**1d**)

According to the procedure of tetrol **1b**, using tetramethyl ether **6d** (25 mg, 0.065 mmol) in CH_2_Cl_2_ (2 mL) and a solution of BBr_3_ in CH_2_Cl_2_ (1 M, 390 μL, 0.39 mmol, 6 equiv) followed by quenching with MeOH and removal of the volatiles gave dipropyltetrol **1c** (19 mg, 0.058 mmol, 89%) as a brown powder: IR (neat) 3252 (br), 2957, 2928, 2868 cm^–1^; ^1^H NMR (400 MHz, CD_3_OD) δ 6.57 (s, 2H), 3.35 (s, 4H), 2.96–2.88 (m, 2H), 2.58–2.51 (m, 2H), 2.49–2.27 (m, 4H), 1.45–1.37 (m, 2H), 1.19–1.06 (m, 2H), 0.48 (t, *J* = 7.4 Hz, 6H) ppm; ^13^C NMR (100 MHz, CD_3_OD) δ 143.9, 142.8, 133.4, 129.0, 128.4, 112.3, 32.6, 31.6, 23.8, 13.7 ppm; HRMS (CI^+^, Orbitrap) *m*/*z* 329.1754 [M + H]^+^ (calcd for C_20_H_25_O_4_, 329.1747).

## Notes

NMR spectra and X-ray diffraction and computational FAIR data are available at the Imperial College research data repository^20^ at DOI: 10.14469/hpc/10703.

## Supporting Information Available

General experimental; copies of ^1^H and ^13^C spectra for all new compounds; comparison of ^1^H NMR resonances for the dimethylene bridges of 4,5-disubstituted 9,10-phenanthrenes **6b**–**d** and **1b**–**d** in the region 3.00–2.00 ppm; analysis of the AA′XX′ “half-spectrum” multiplet at δ = 153.4 ppm for the fluorine-bearing carbons in the ^13^C NMR spectrum; X-ray crystal data for **4b**, **5c**, **6a**–**d**, and **7c** (CCDC 1947920–1947926); CSP-HPLC chromatograms for **6a**–**d** (PDF)
