# Supplementary material for: (±)-Polysiphenol and Other Analogues via Symmetrical Intermolecular Dimerizations: A Synthetic, Spectroscopic, Structural, and Computational Study
Source: J Nat Prod. 2022 Oct 26;85(11):2650–5. doi: 10.1021/acs.jnatprod.2c00749 (PMC9706781; doi:10.1021/acs.jnatprod.2c00749)
Supplement: Supplementary file 1 — np2c00749_si_001.pdf [file np2c00749_si_001.pdf]

SUPPORTING INFORMATION

(±)-Polysiphenol and other Analogues via Symmetrical Intermolecular Dimerizations: A Synthetic, Spectroscopic, Structural and Computational Study.

D. Christopher Braddock,* Anna Duran-Corbera, Masih Nilforoushan, Ziye Yang, Tianyou He, Gajan Santhakumar, Karim A. Bahou, Henry S. Rzepa, Rudiger Woscholski and Andrew J. P. White

Department of Chemistry, Molecular Sciences Research Hub, Imperial College London, White City Campus, 82 Wood Lane, London W12 0BZ, UK

Email Address: c.braddock@imperial.ac.uk

Cover Page and Contents

pS1	Cover page and contents;
pS2	General experimental;
pS3-22	Copies of ^{1}H and ^{13}C spectra for all new compounds;
pS23	Comparison of ^{1}H NMR resonances for the dimethylene bridges of 4,5-disubstituted 9,10-phenanthrenes **6b-d** and **1b-d** in the region 3.00-2.00 ppm;
pS24	Analysis of the AA'XX' 'half-spectrum' multiplet at δ = 153.4 ppm for the fluorine-bearing carbons in the ^{13}C NMR spectrum of **6c**;
pS25-31	X-Ray Crystal Data for **4b**, **5c**, **6a-d** and **7c** (CCDC 1947920-1947926);
pS32	CSP-HPLC chromatograms for **6a-d**;
pS33	SI references.

General Experimental

Reagents: All reagents were purchased from commercial suppliers and used as received.

Solvents: Anhydrous tetrahydrofuran and dichloromethane were obtained from a purification column composed of activated alumina and were used directly. DMF, DMSO, extraction solvents and solvents used in column chromatography were used as received at HPLC grade.

Experimental techniques: All synthetic procedures were used or adapted from literature and were conducted in oven-dried glassware under an inert atmosphere of nitrogen, unless otherwise stated. Reaction temperatures other than room temperature were recorded either as oil bath or cooling bath temperature. All volatiles and compounds were removed/concentrated *in vacuo* by rotary evaporation or under reduced pressure (Schlenck line). Kieselgel 60 F254 pre-coated aluminium-backed plates were used for analytical Thin Layer Chromatography and visualized either using UV light (254 or 350 nm) or by chemical staining with potassium permanganate or vanillin. Flash column chromatography was performed using Geduran® silica gel, particle size 40-63 μm.

Characterisation: Melting points were determined using a Lambda Photometrics MPA100 OptiMelt melting point device or Stuart Melting Point Apparatus (SMP10) and are uncorrected. An ATR-IR spectrometer was used to obtain FT-IR spectra of neat compounds unless otherwise stated. ^{1}H NMR (400 MHz) spectra, ^{13}C NMR (100 MHz) and ^{19}F NMR (376 MHz) spectra were recorded in $CDCl_3$ at 298 K unless otherwise stated using the NMR facility at Imperial College London on either a Bruker DRX-400 or Bruker AV-400. Chemical shifts (δ) are quoted in parts per million (ppm) and are downfield relative to tetramethylsilane ($SiMe_4$, δ = 0.00 ppm) and referenced to the residual solvent peak (δ = 7.26 ppm for $CDCl_3$). Abbreviations used for multiplicity are as follows: s – singlet, d – doublet, br – broad, m – multiplet. Low resolution MS (CI and EI) and high resolution MS were recorded by the Imperial College Department of Chemistry Mass Spectrometry Service. X-Ray Crystallography studies were conducted on suitable single crystals, by the Imperial College Department of Chemistry X-Ray Crystallography Facility.

Copies of ^{1}H and ^{13}C spectra for all new compounds

4,4'-(Ethene-1,2-diyl)bis(2-bromo-6-methoxyphenol) (3a)

^{1}H NMR Spectrum (400 MHz, DMSO-d_6)

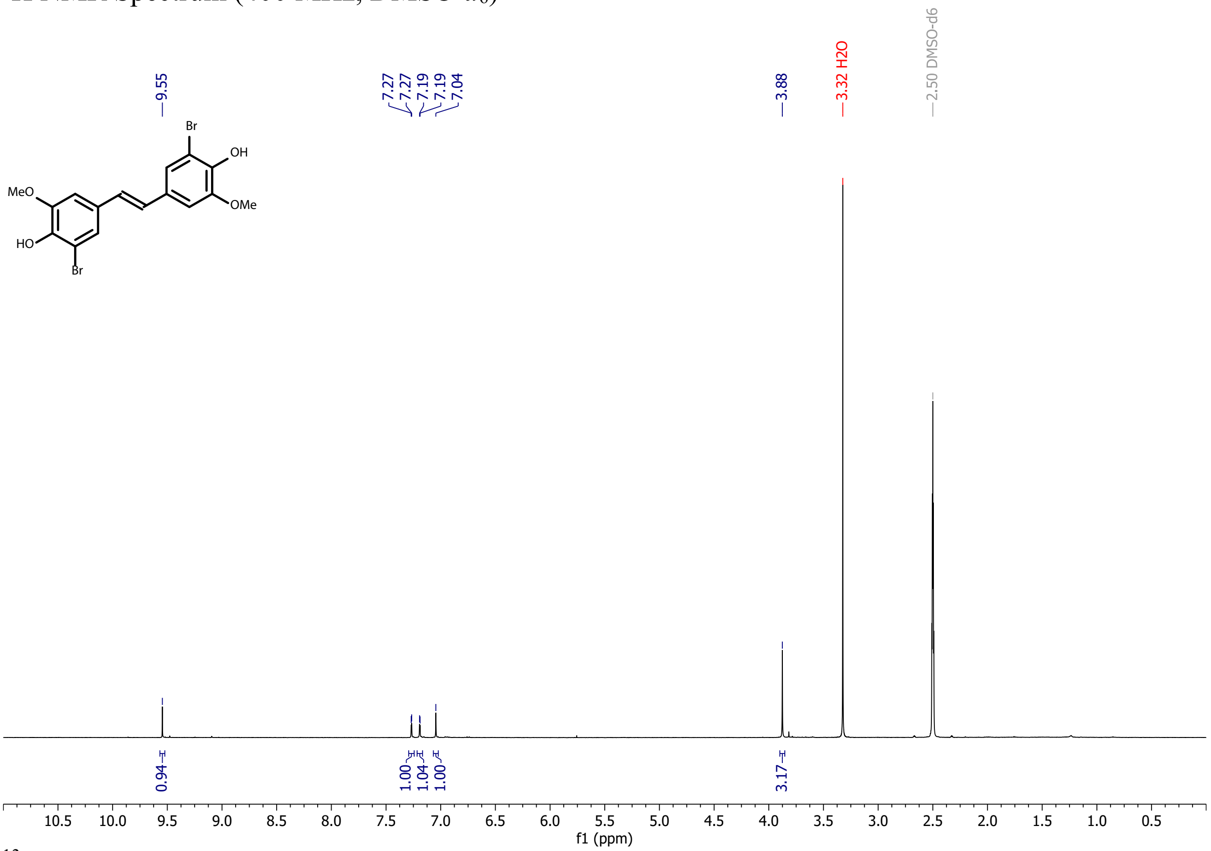

^{13}C NMR Spectrum (100 MHz, DMSO-d_6)

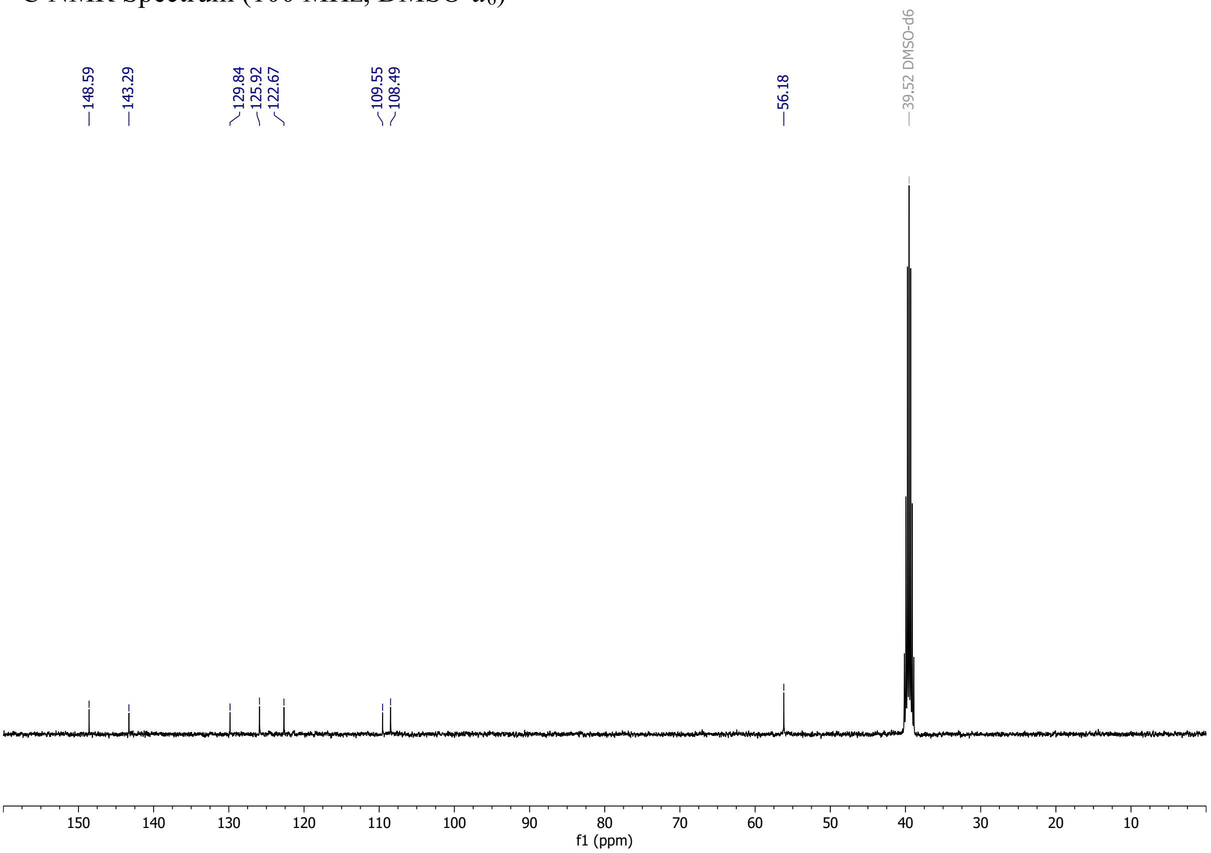

4,4'-(Ethane-1,2-diyl)bis(2-bromo-6-methoxyphenol) (4a)

^{1}H NMR Spectrum (400 MHz, DMSO-d_6)

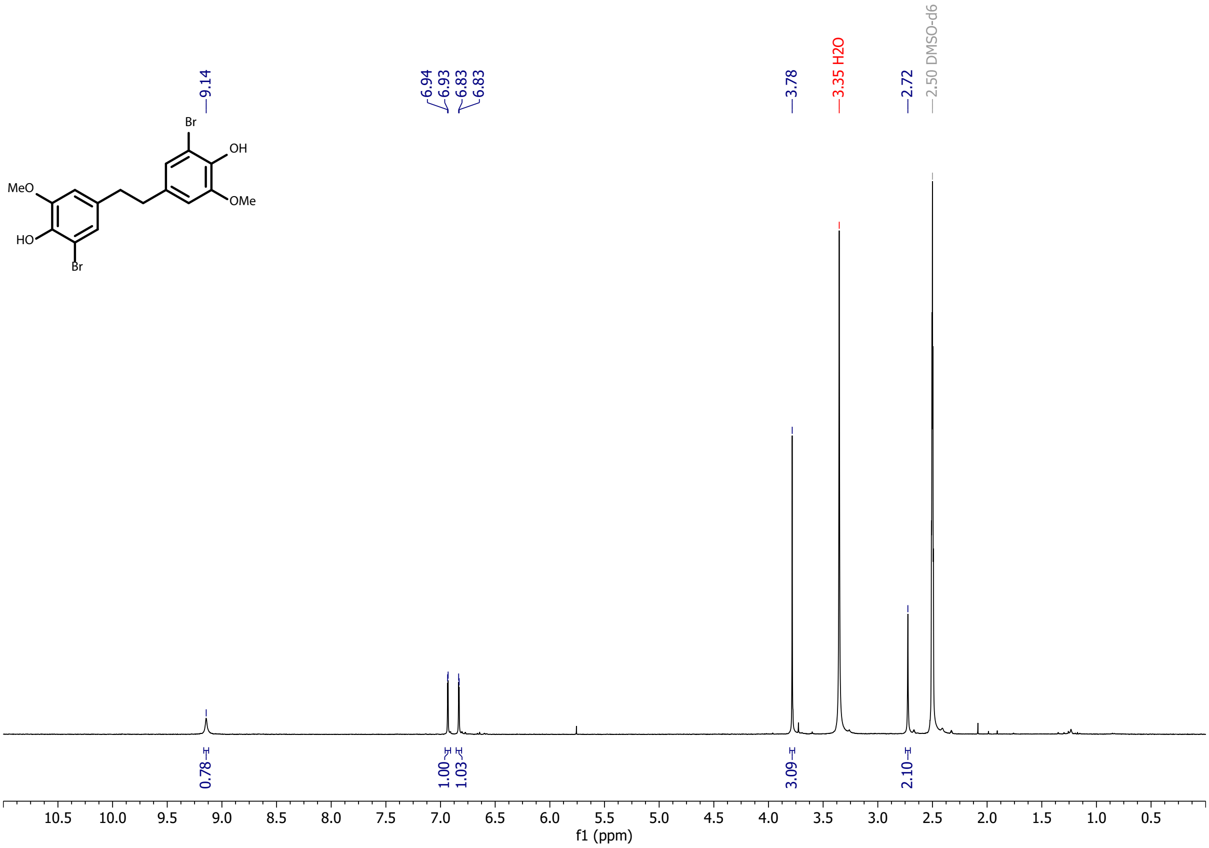

^{13}C NMR Spectrum (100 MHz, DMSO-d_6)

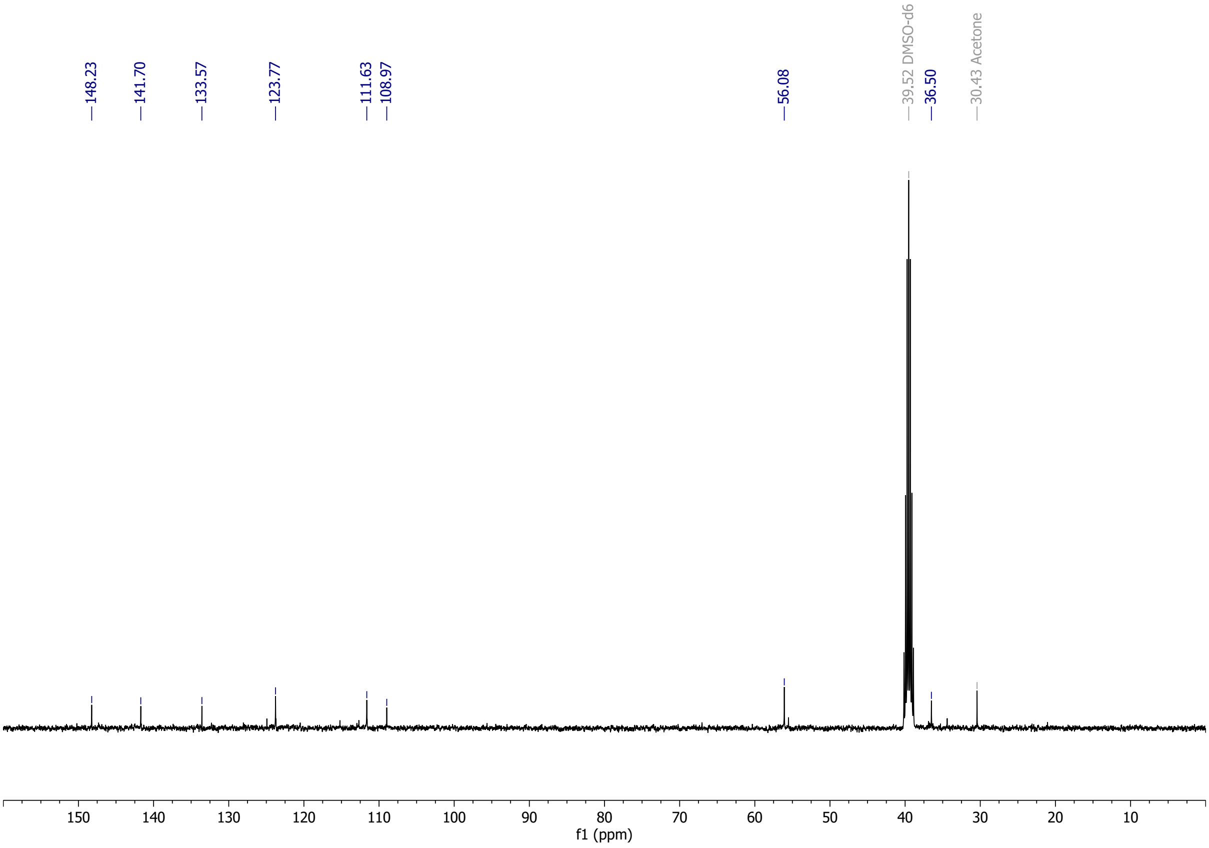

1,2-Bis(3-bromo-4,5-dimethoxyphenyl)ethane (5a)

^{1}H NMR Spectrum (400 MHz, $CDCl_3$)

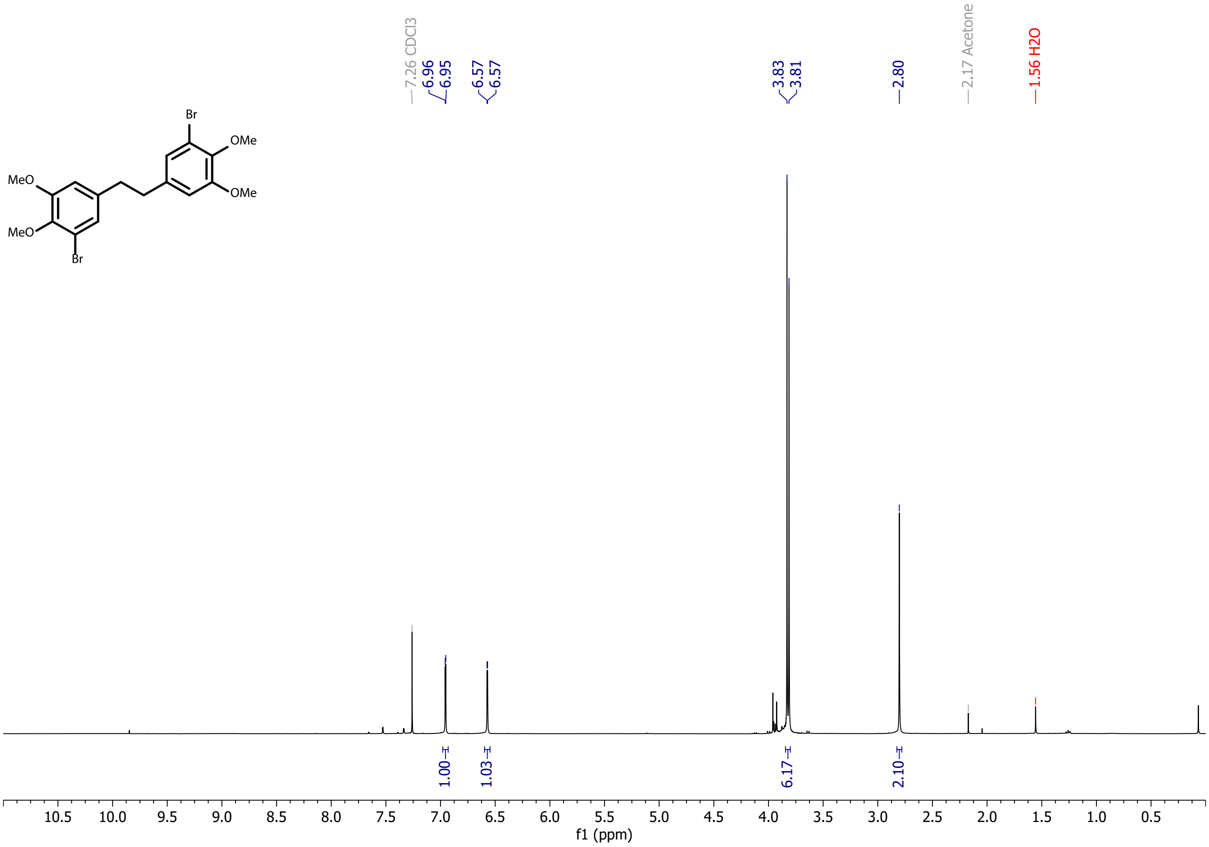

^{13}C NMR Spectrum (100 MHz, $CDCl_3$)

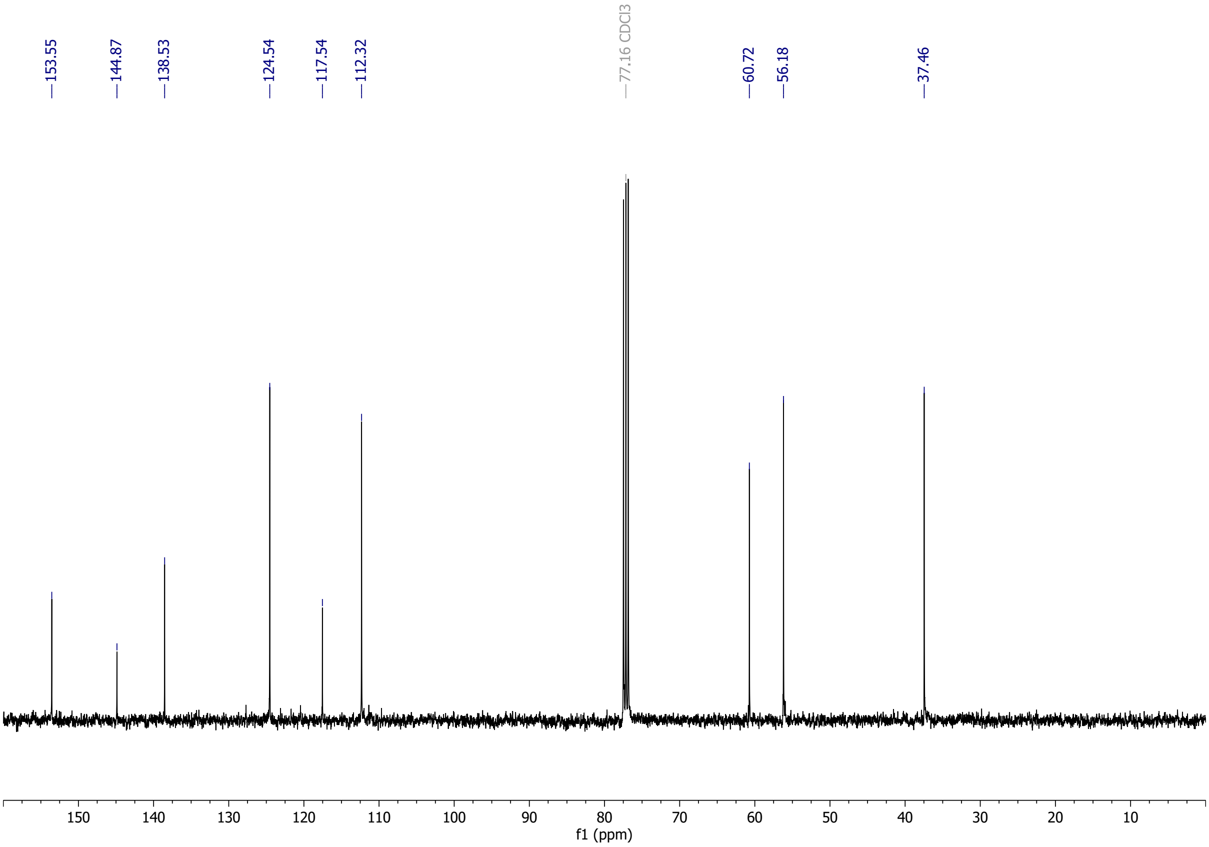

4,4'-(Ethene-1,2-diyl)bis(2-chloro-6-methoxyphenol) (3b)

^{1}H NMR Spectrum (400 MHz, DMSO-d_6)

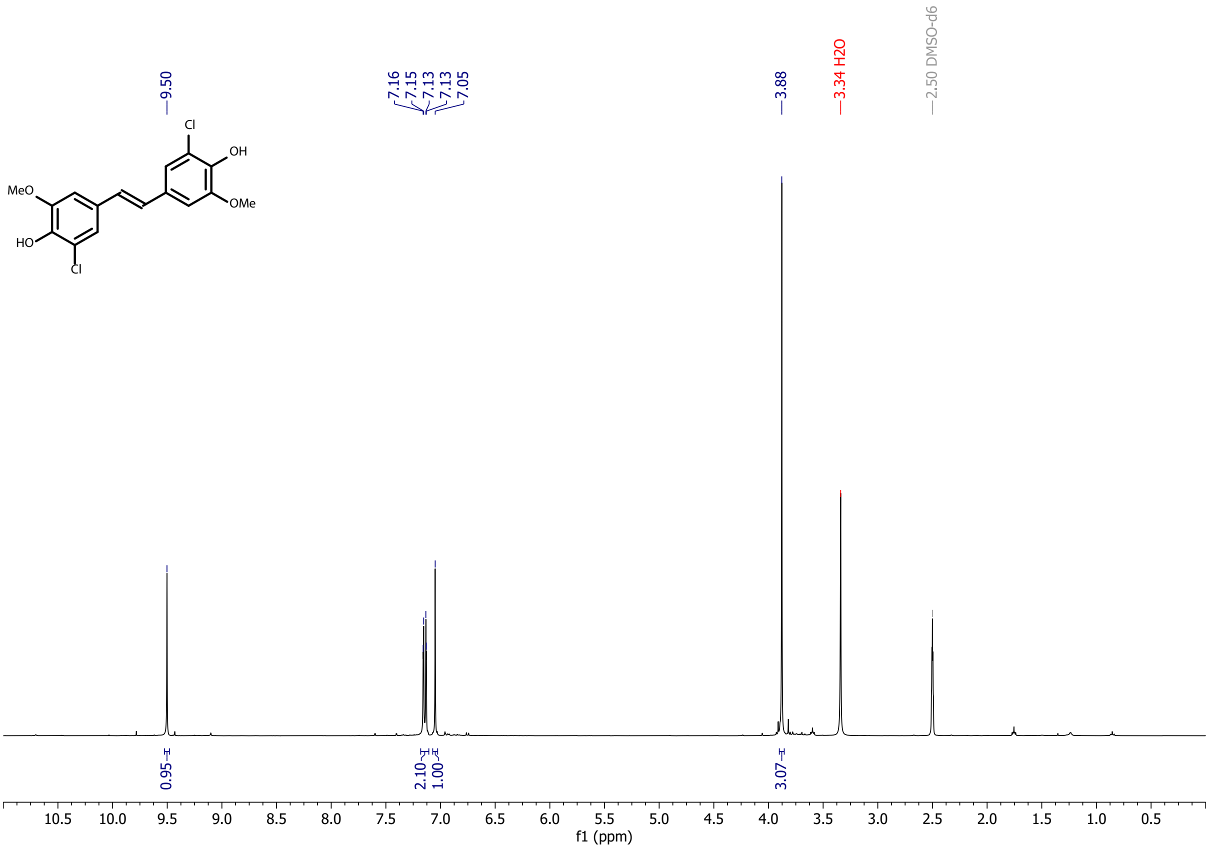

^{13}C NMR Spectrum (100 MHz, DMSO-d_6)

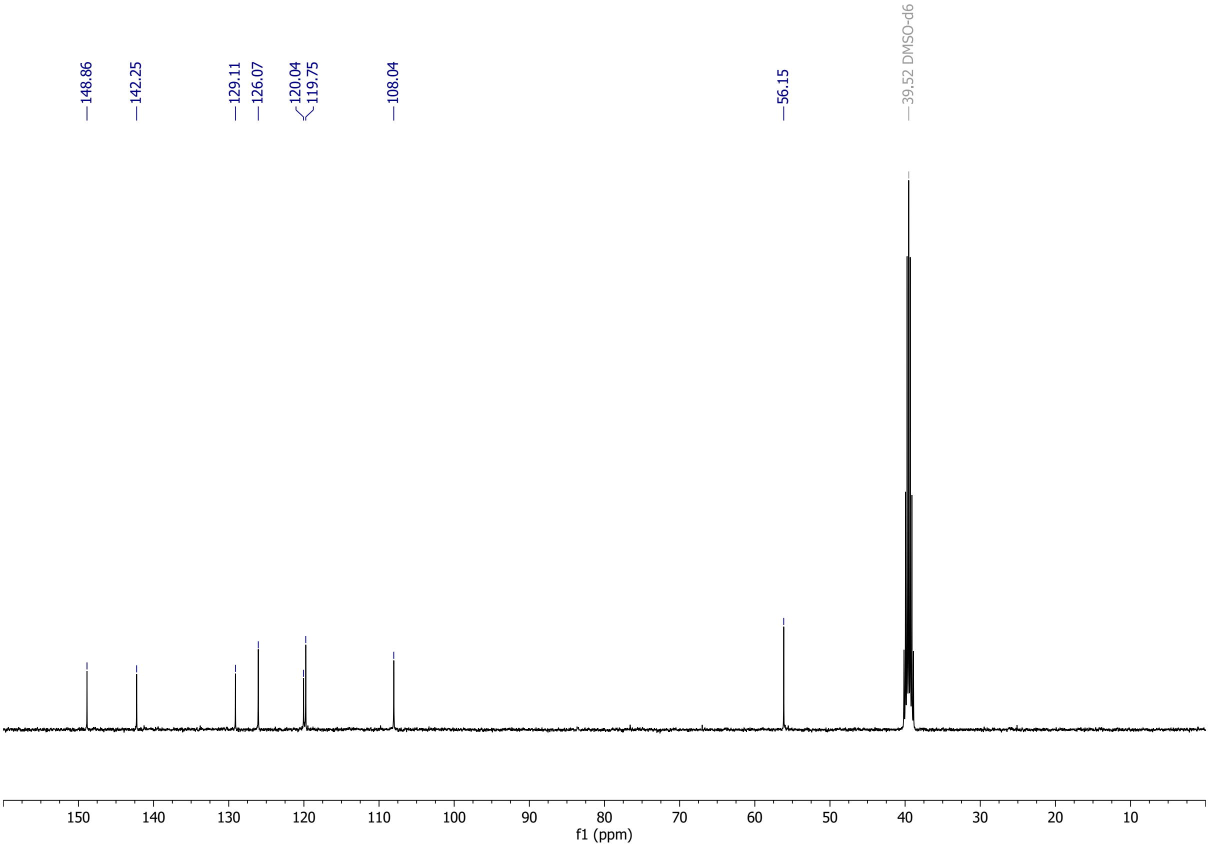

4,4'-(Ethane-1,2-diyl)bis(2-chloro-6-methoxyphenol) (4b)

^{1}H NMR Spectrum (400 MHz, DMSO-d_6)

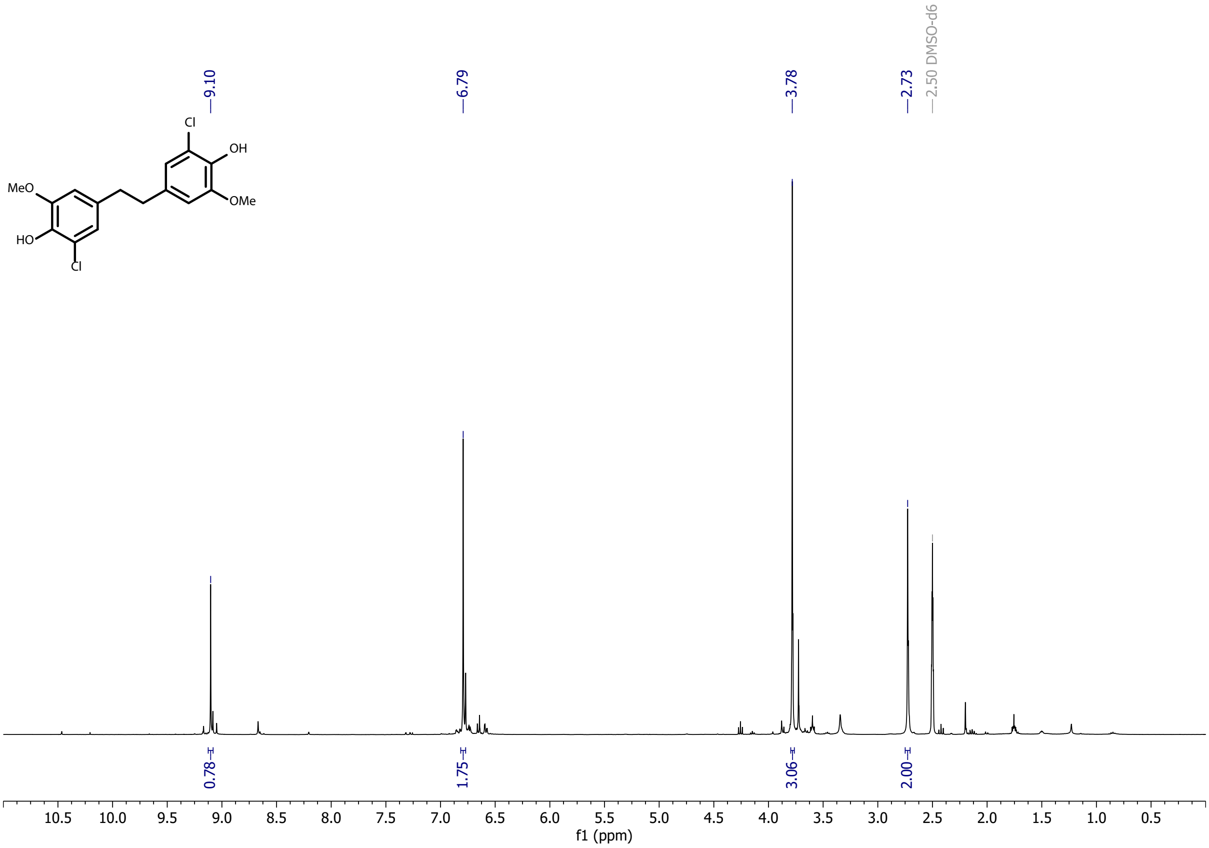

^{13}C NMR Spectrum (100 MHz, DMSO-d_6)

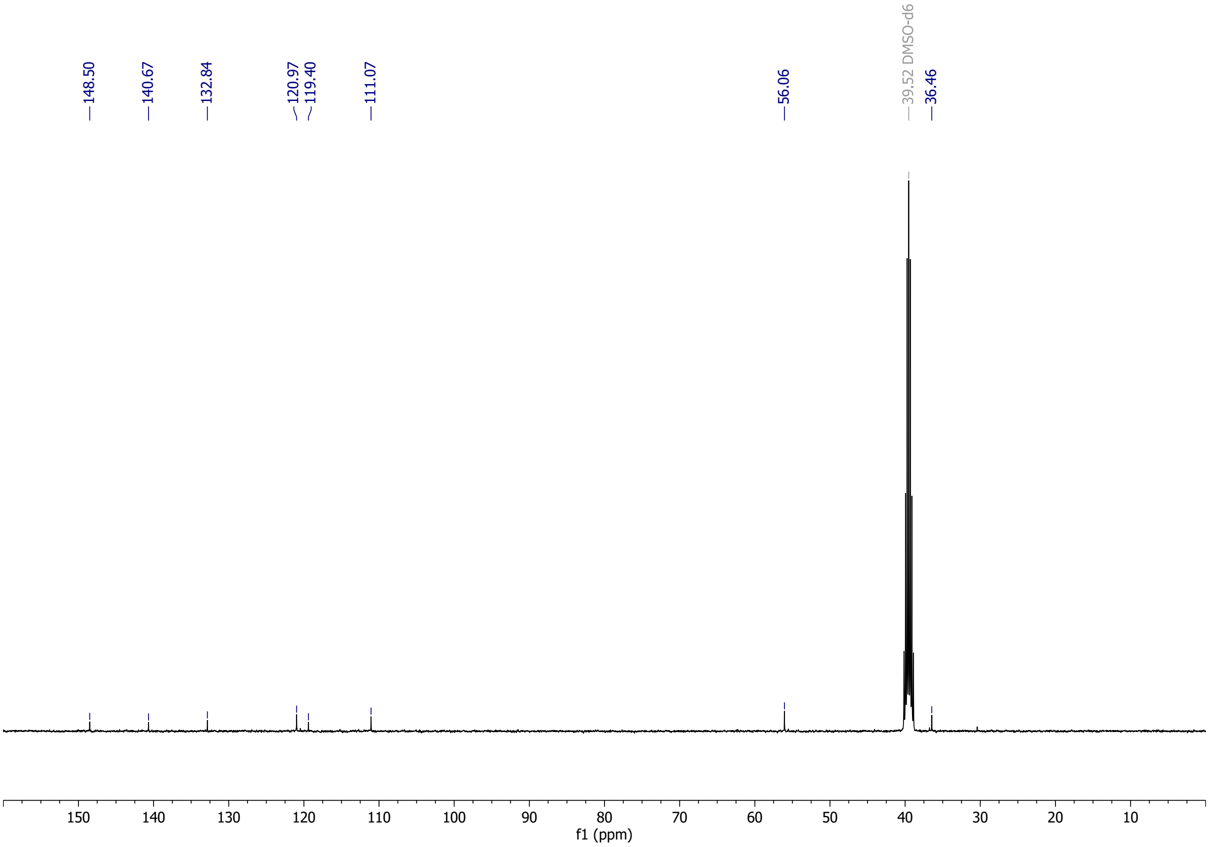

1,2-Bis(3-chloro-4,5-dimethoxyphenyl)ethane (5b)

^{1}H NMR Spectrum (400 MHz, $CDCl_3$)

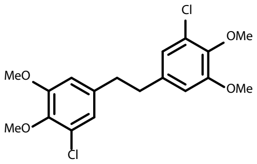

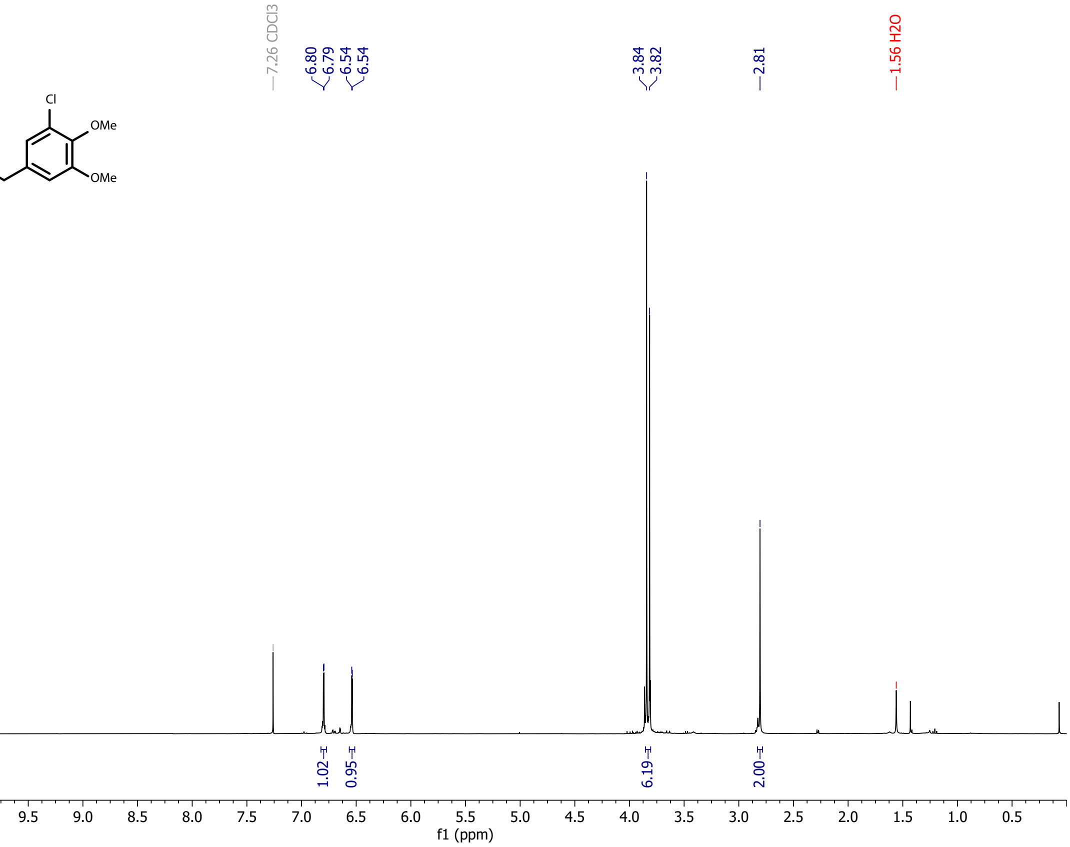

^{13}C NMR Spectrum (100 MHz, $CDCl_3$)

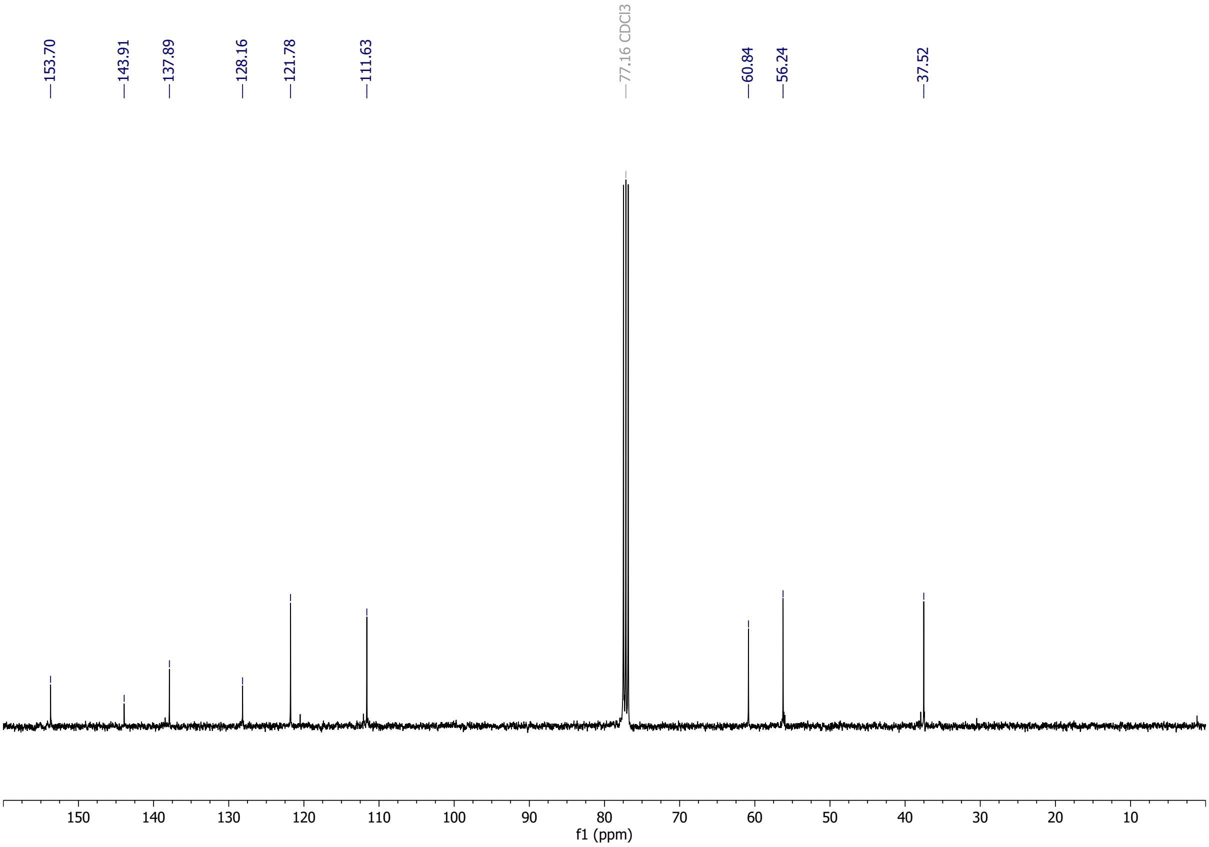

4,5-Dichloro-2,3,6,7-tetramethoxy-9,10-dihydrophenanthrene (6b)

^{1}H NMR Spectrum (400 MHz, $CDCl_3$)

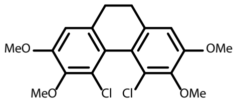

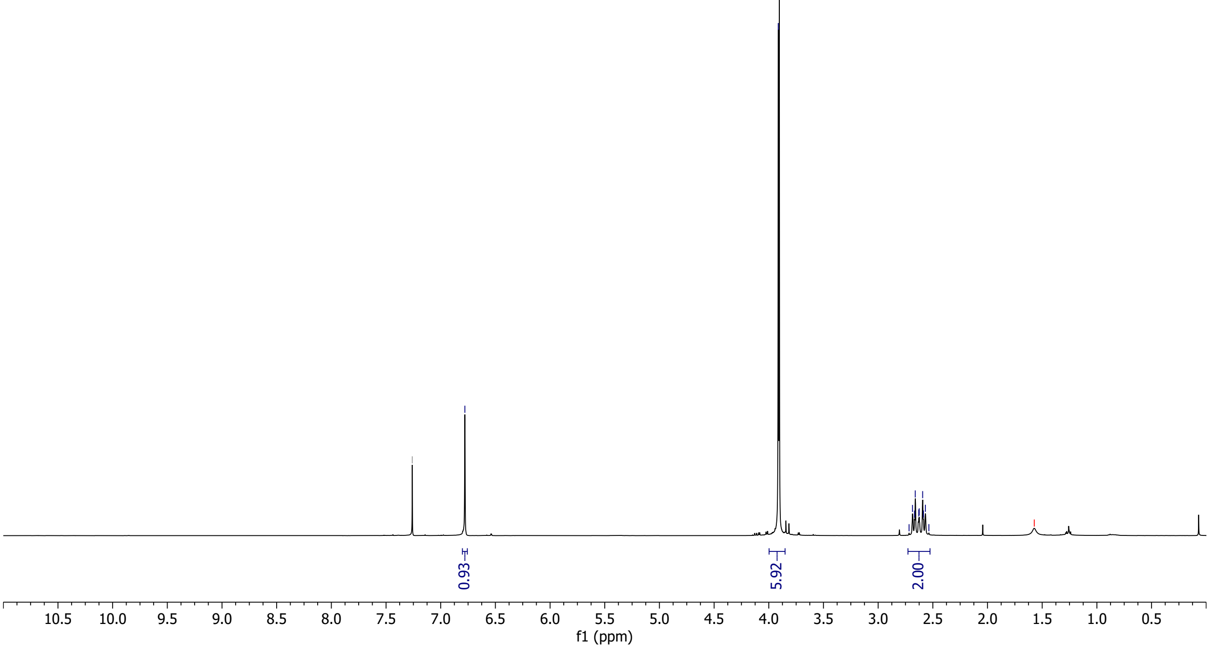

^{13}C NMR Spectrum (100 MHz, $CDCl_3$)

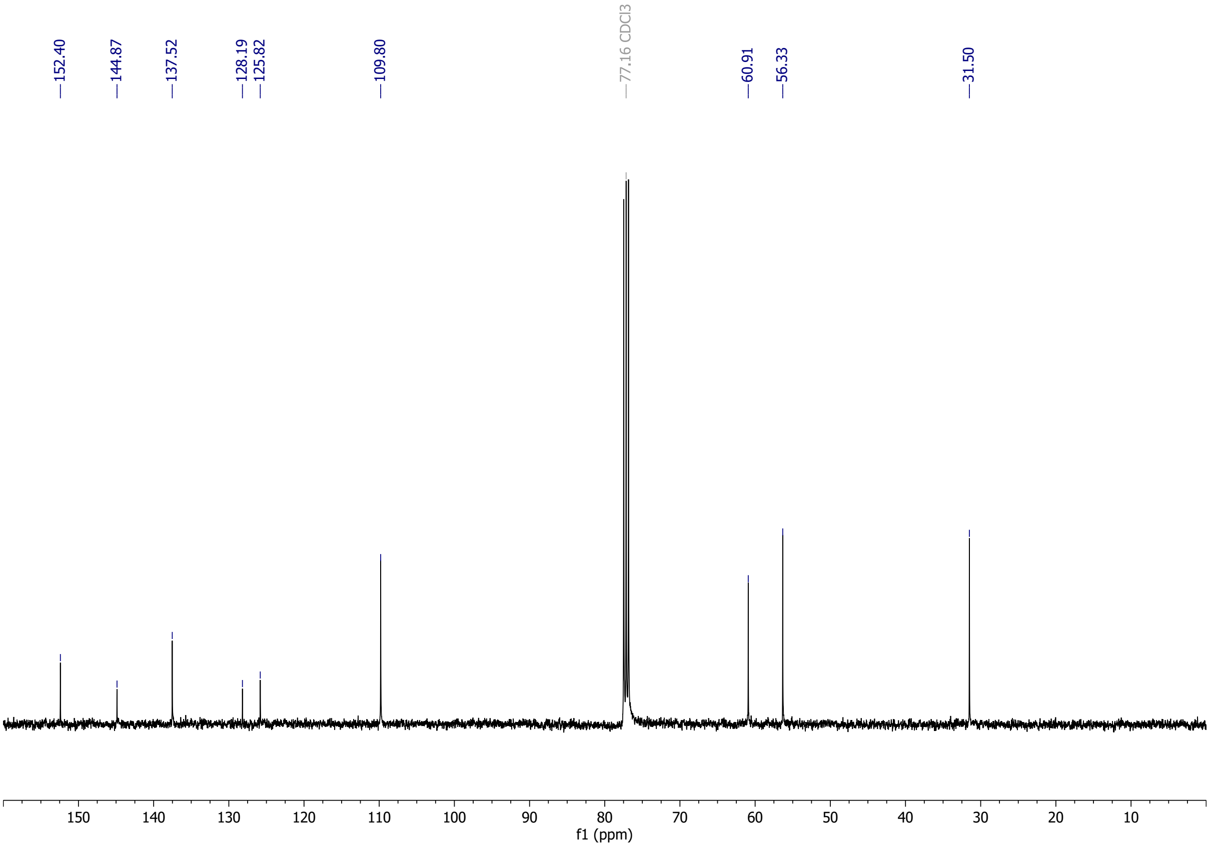

4,5-Dichloro-9,10-dihydrophenanthrene-2,3,6,7-tetraol (1b)

^{1}H NMR Spectrum (400 MHz, CD_3OD)

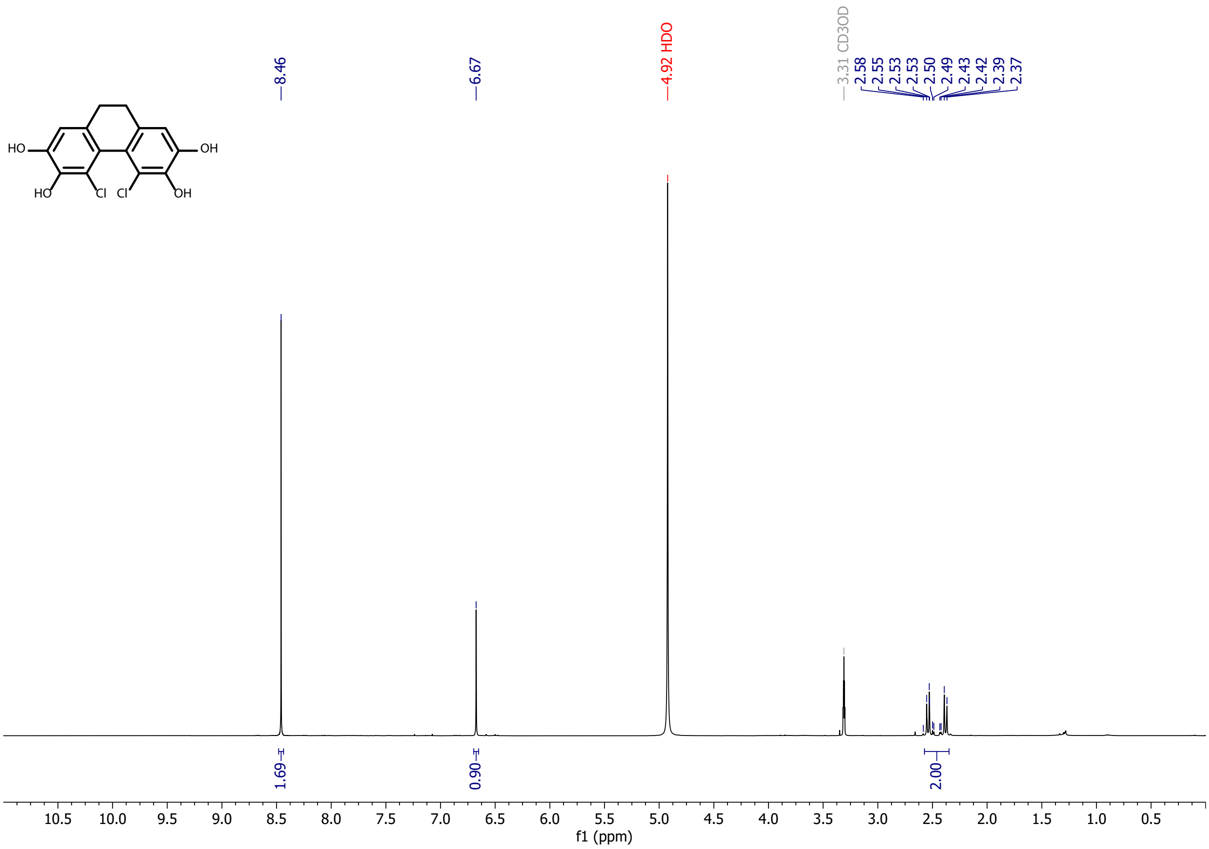

^{13}C NMR Spectrum (100 MHz, DMSO-d_6)

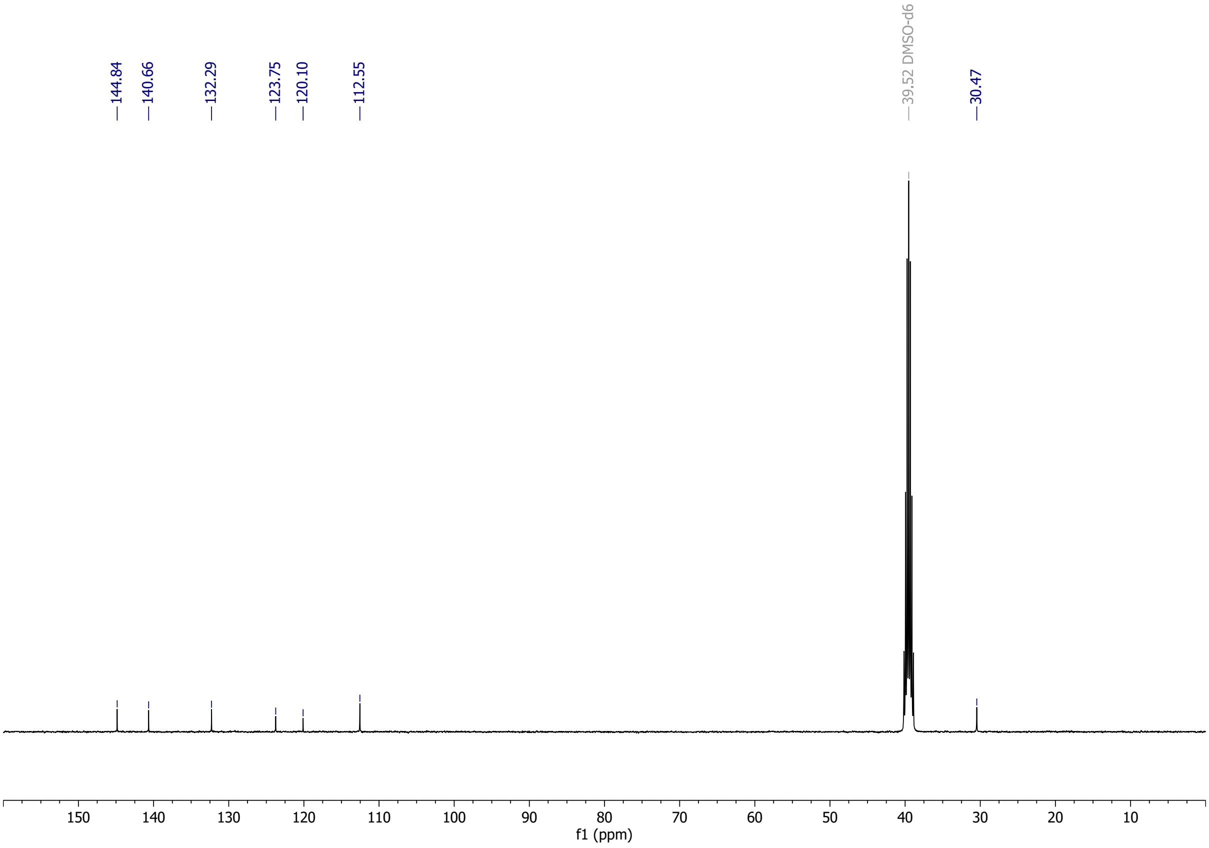

4,4'-(Ethene-1,2-diyl)bis(2-fluoro-6-methoxyphenol) (3c)

^{1}H NMR Spectrum (400 MHz, DMSO-d_6)

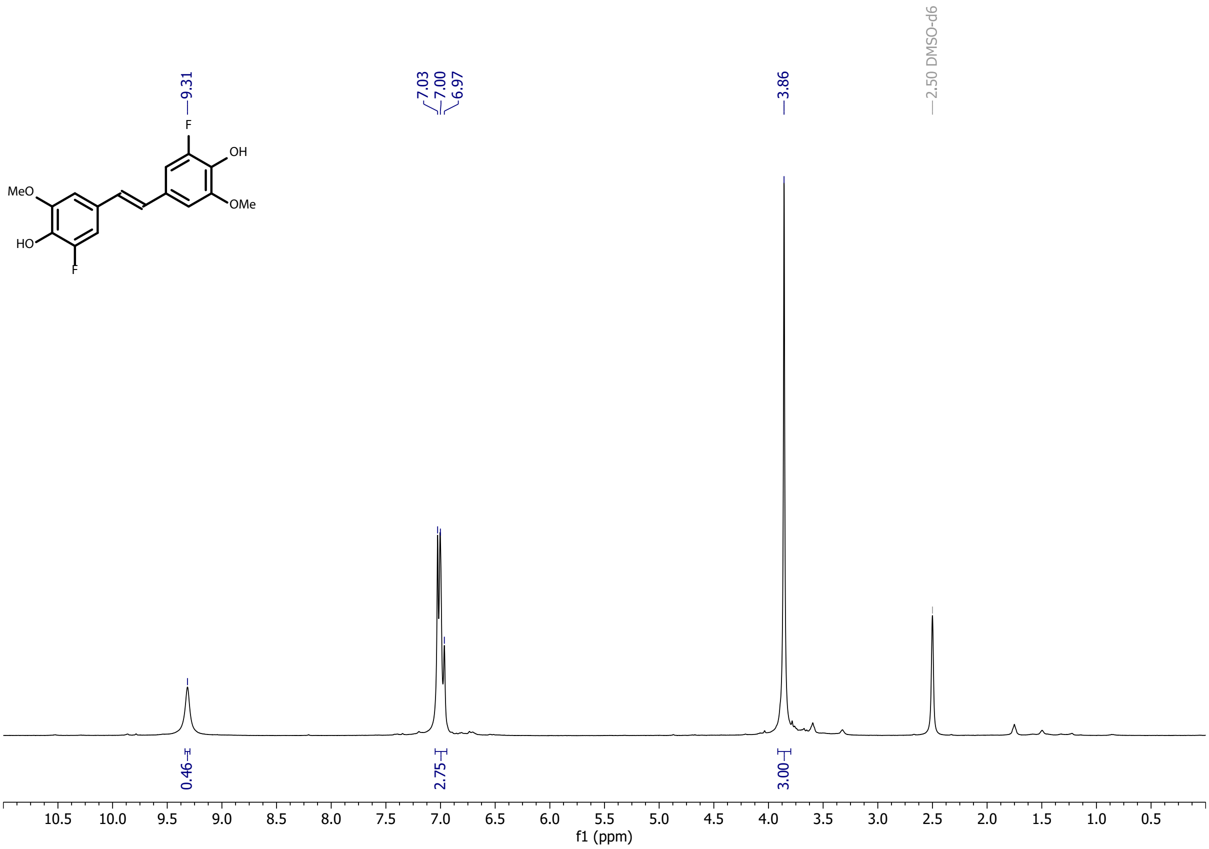

^{13}C NMR Spectrum (100 MHz, DMSO-d_6)

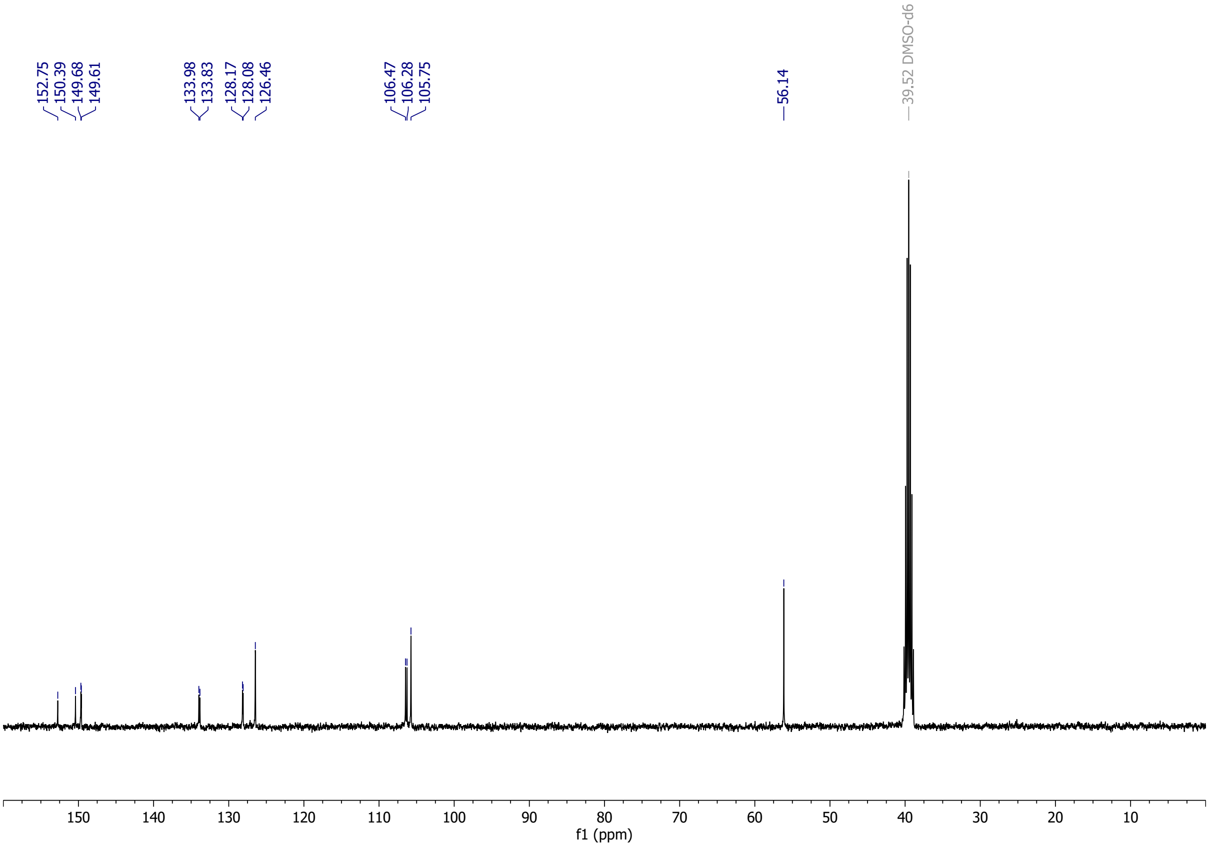

4,4'-(Ethane-1,2-diyl)bis(2-fluoro-6-methoxyphenol) (4c)

^{1}H NMR Spectrum (400 MHz, DMSO-d_6)

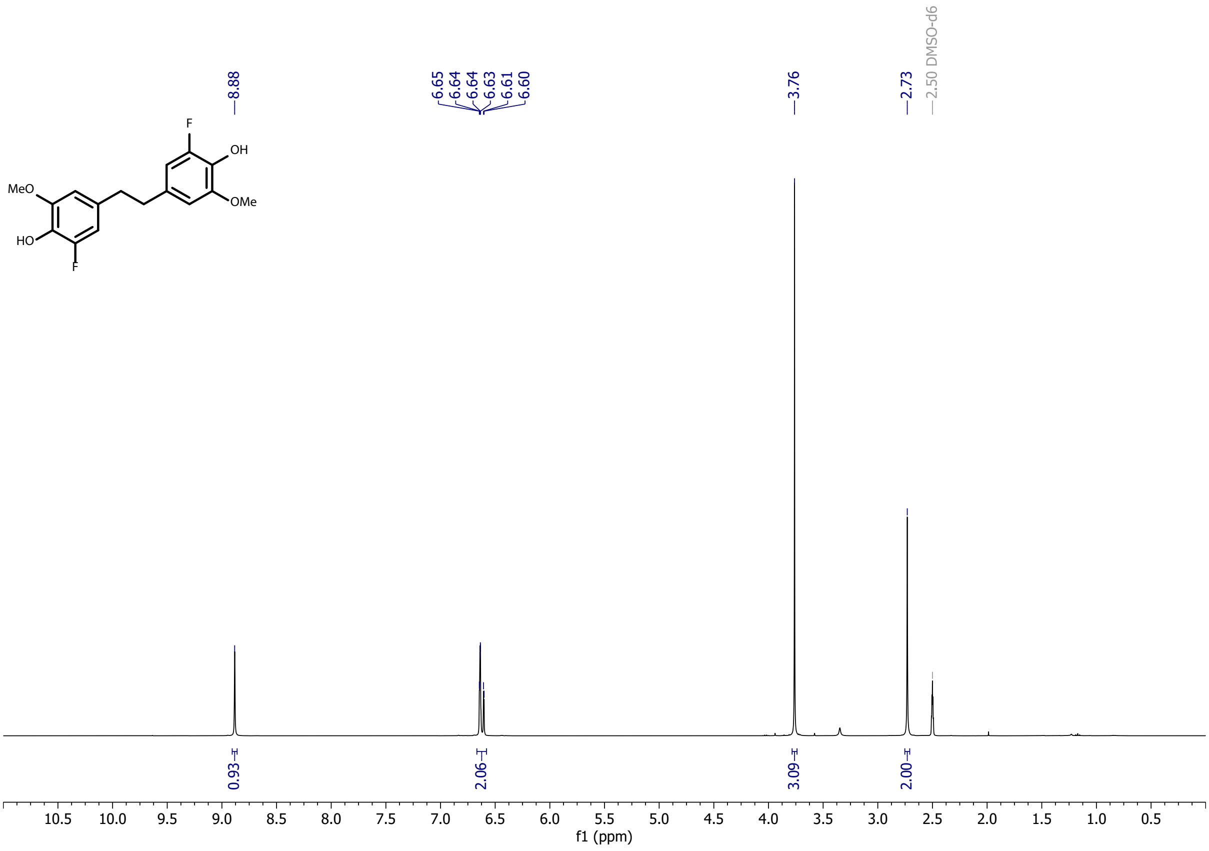

^{13}C NMR Spectrum (100 MHz, DMSO-d_6)

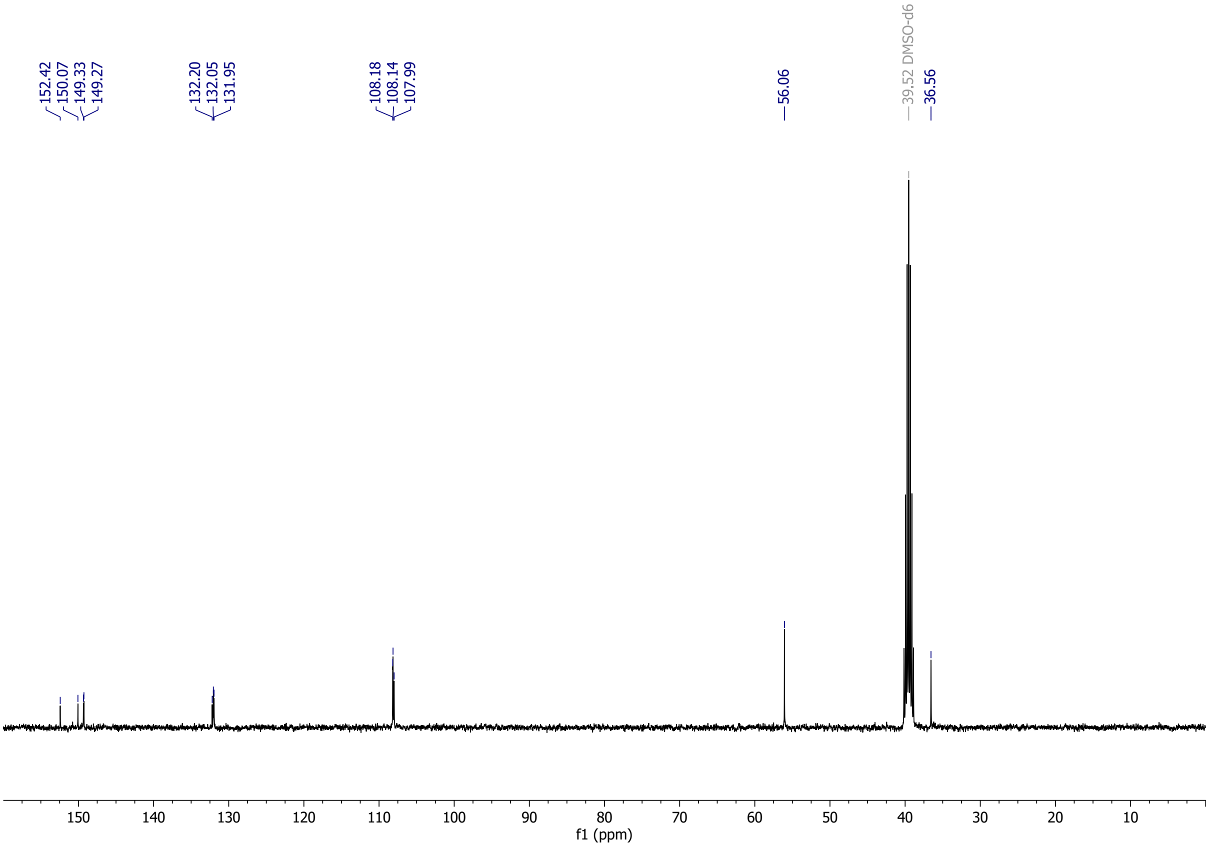

1,2-Bis(3-fluoro-4,5-dimethoxyphenyl)ethane (5c)

^{1}H NMR Spectrum (400 MHz, $CDCl_3$)

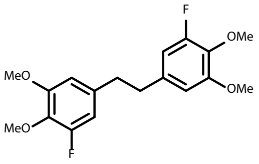

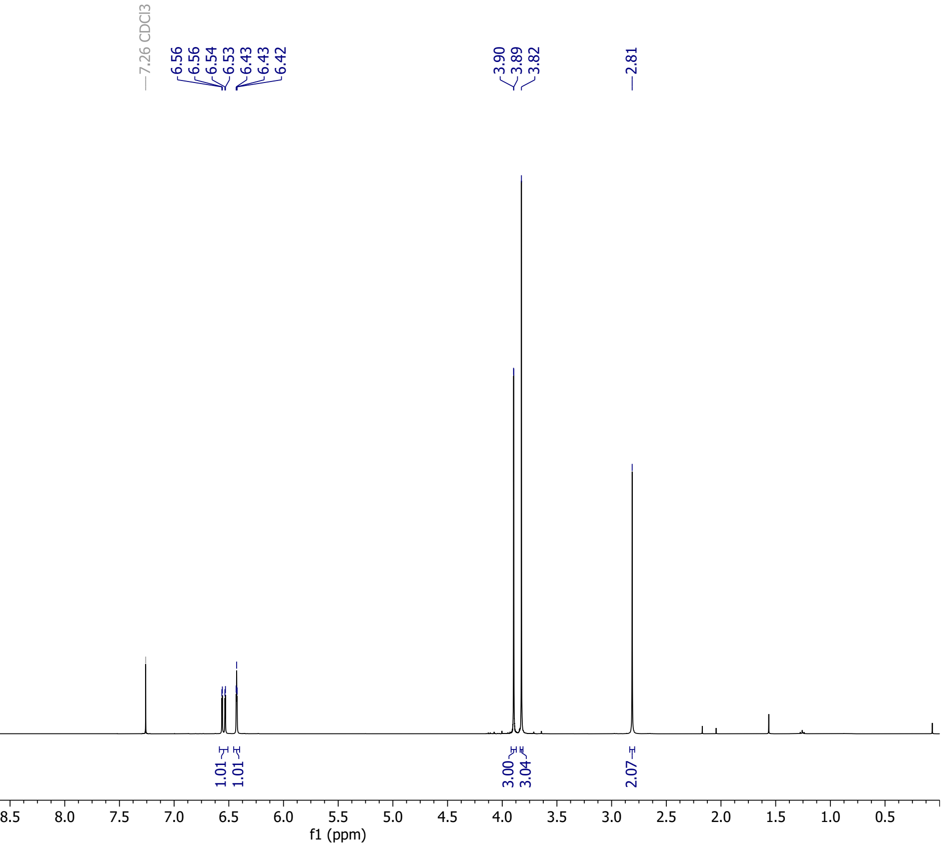

^{13}C NMR Spectrum (100 MHz, $CDCl_3$)

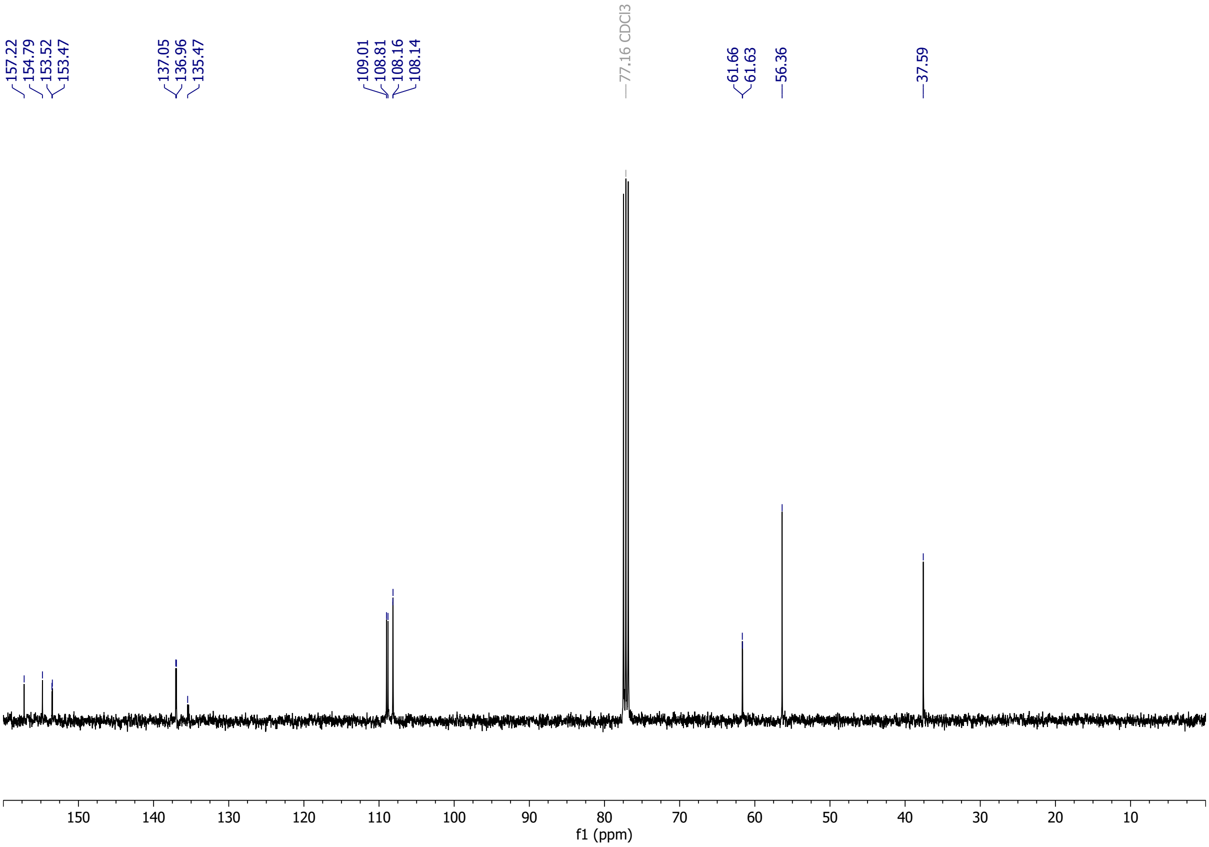

4,5-Difluoro-2,3,6,7-tetramethoxy-9,10-dihydrophenanthrene (6c)

^{1}H NMR Spectrum (400 MHz, $CDCl_3$)

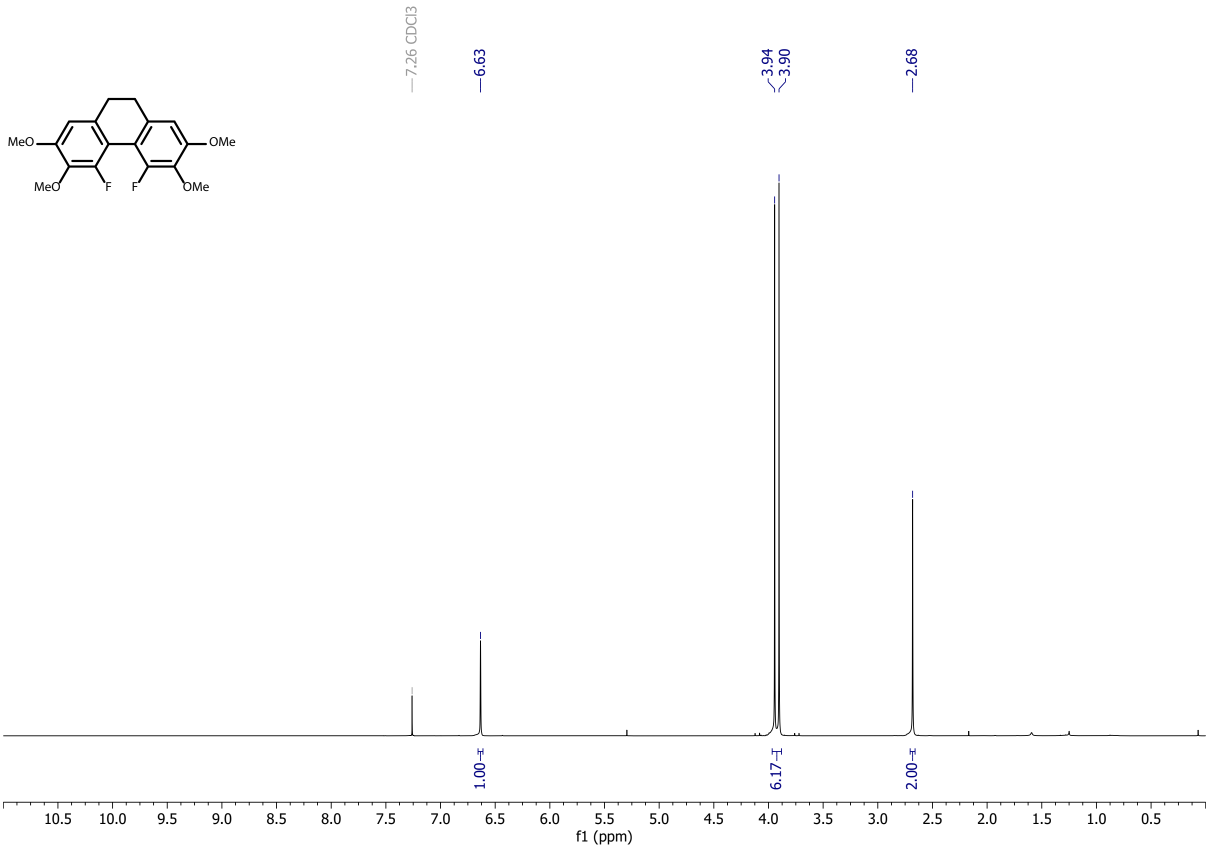

^{13}C NMR Spectrum (100 MHz, $CDCl_3$)

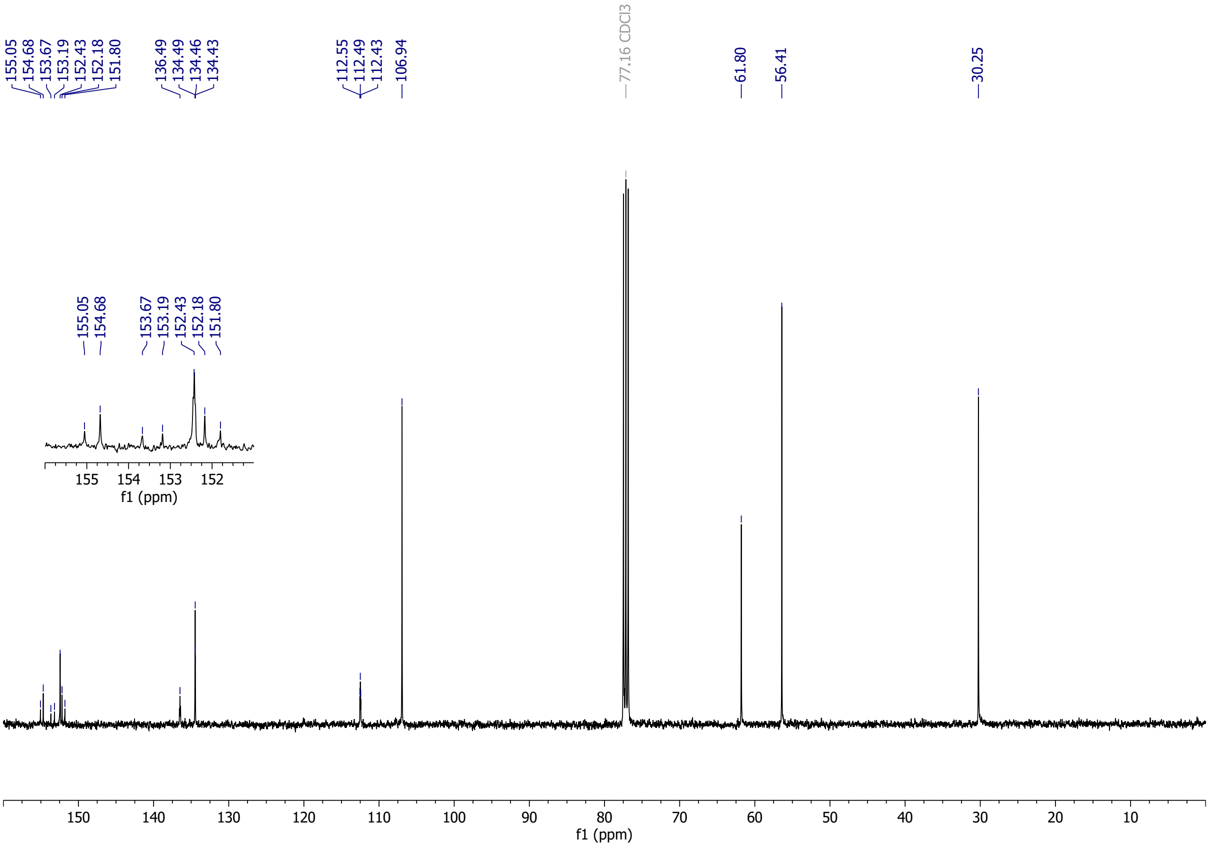

^{13}C NMR Spectrum (100 MHz, $CDCl_3$, ^{19}F Decoupled)

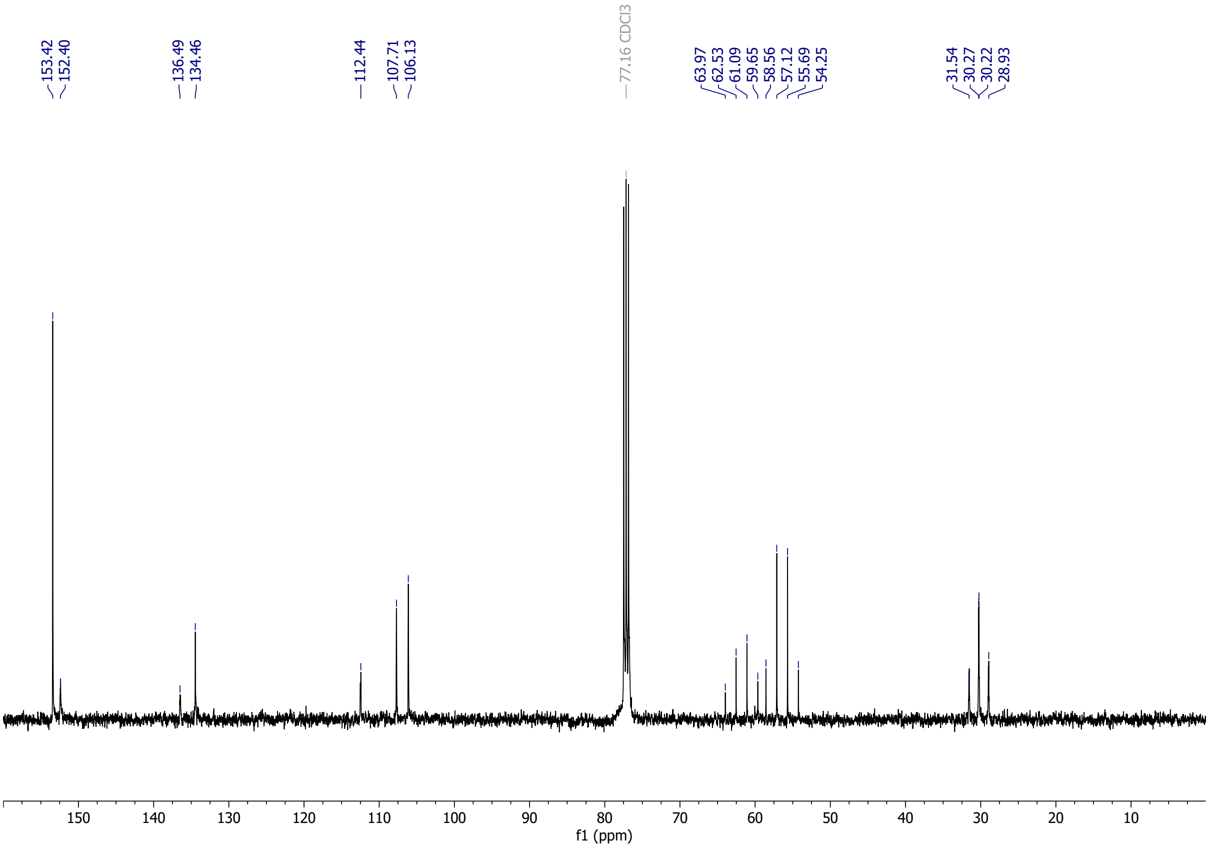

4,5-Difluoro-2,3,6,7-tetramethoxyphenanthrene (7c)

^{1}H NMR Spectrum (400 MHz, $CDCl_3$)

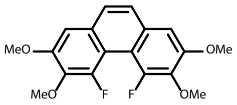

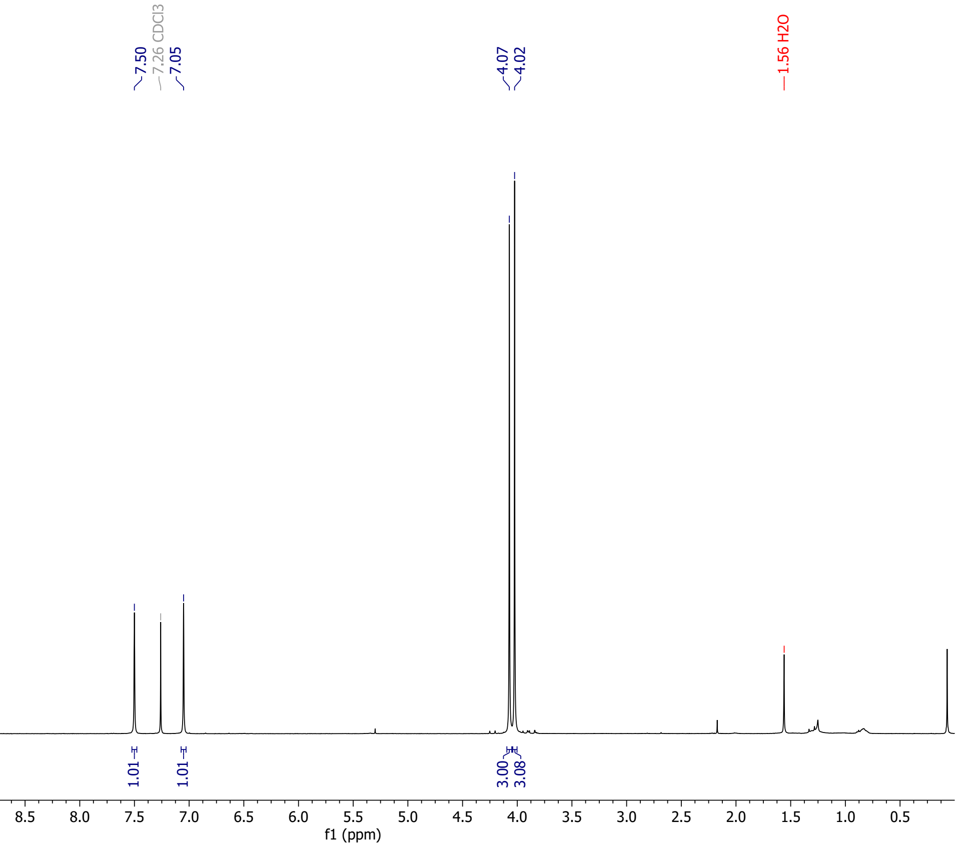

4,5-Difluoro-9,10-dihydrophenanthrene-2,3,6,7-tetraol (1c)

^{1}H NMR Spectrum (400 MHz, CD_3OD)

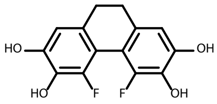

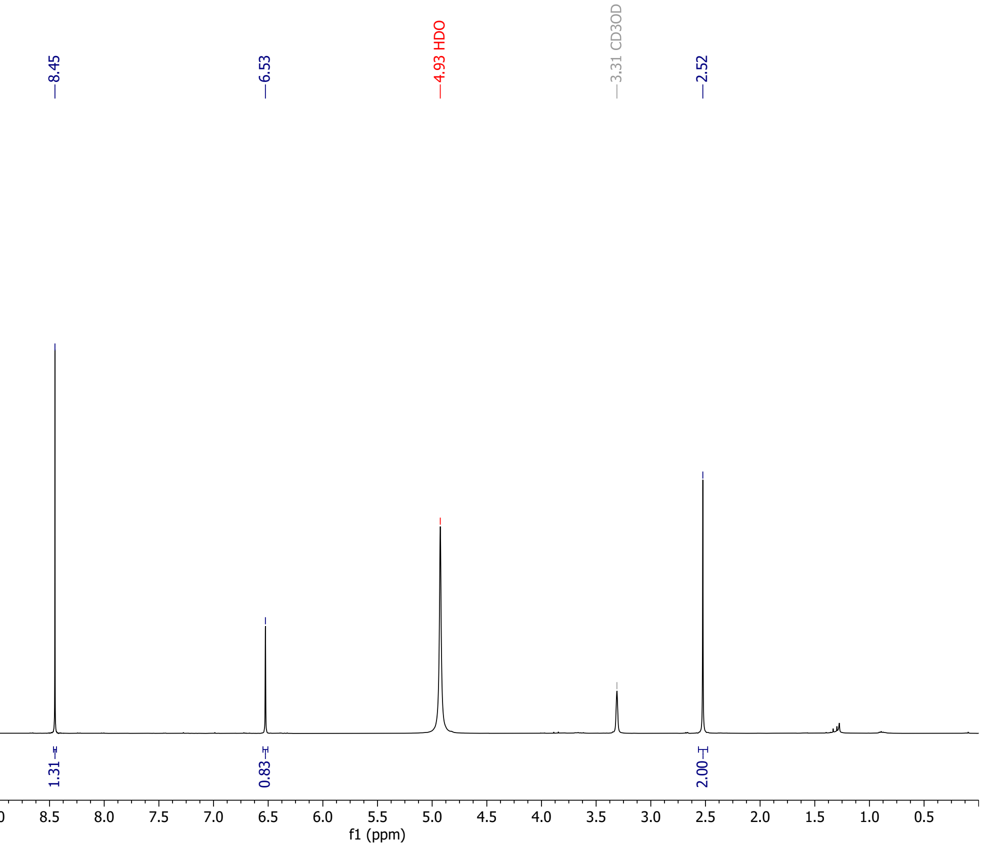

4,4'-(Ethene-1,2-diyl)bis(2-allyl-6-methoxyphenol) (3d)

^{1}H NMR Spectrum (400 MHz, $CDCl_3$)

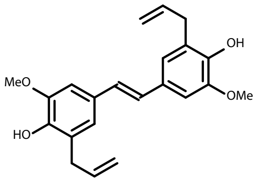

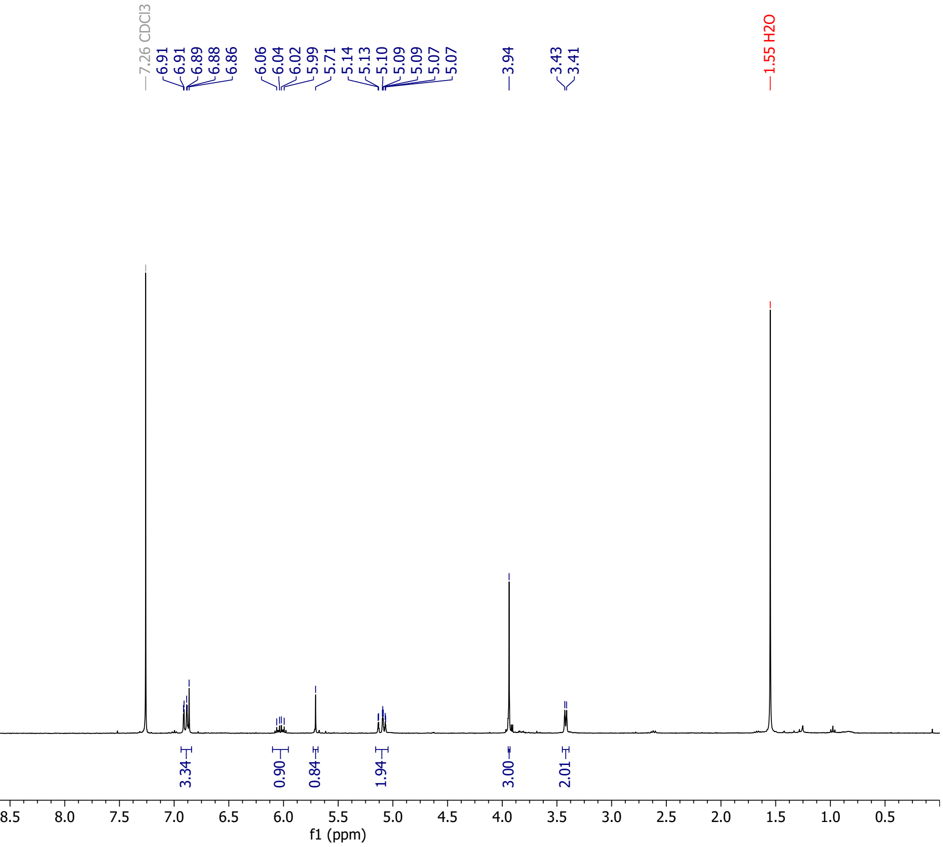

^{13}C NMR Spectrum (100 MHz, $CDCl_3$)

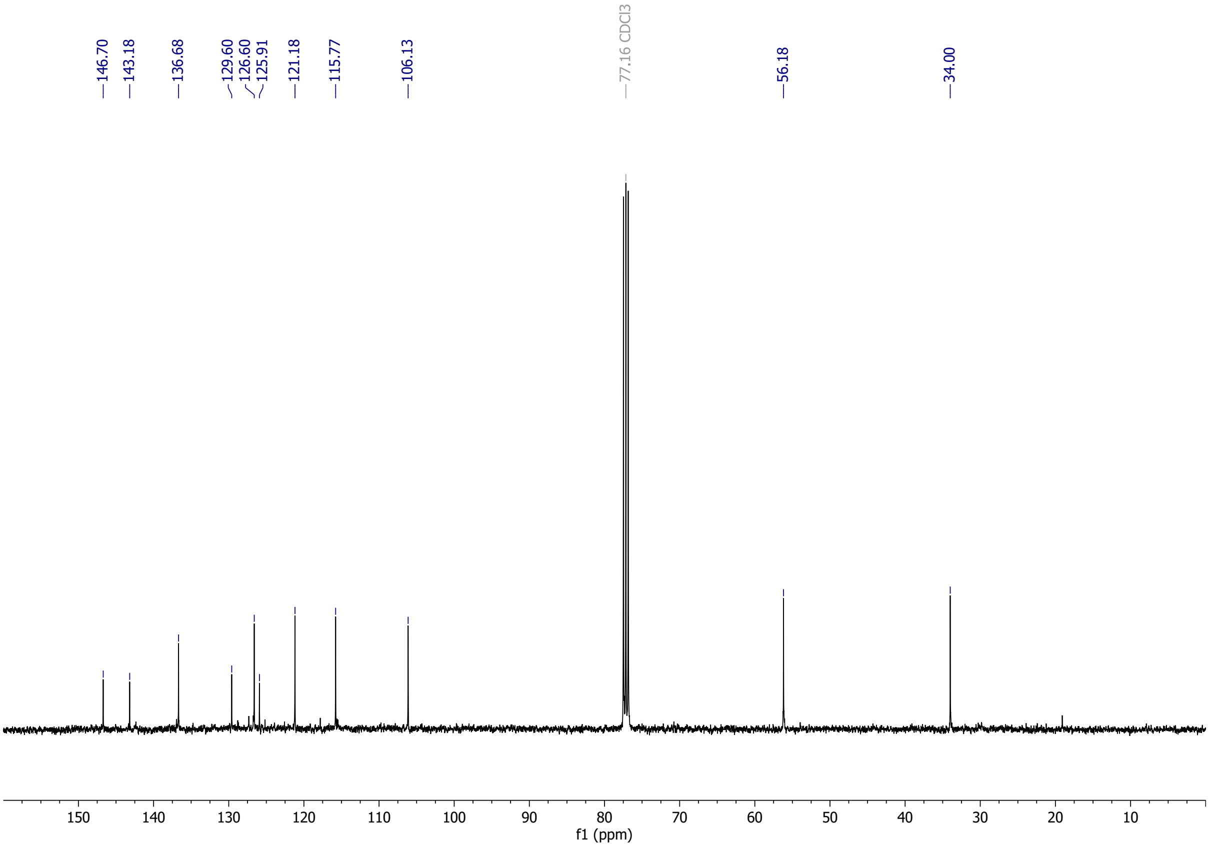

4,4'-(Ethane-1,2-diyl)bis(2-methoxy-6-propylphenol) (4d)

^{1}H NMR Spectrum (400 MHz, $CDCl_3$)

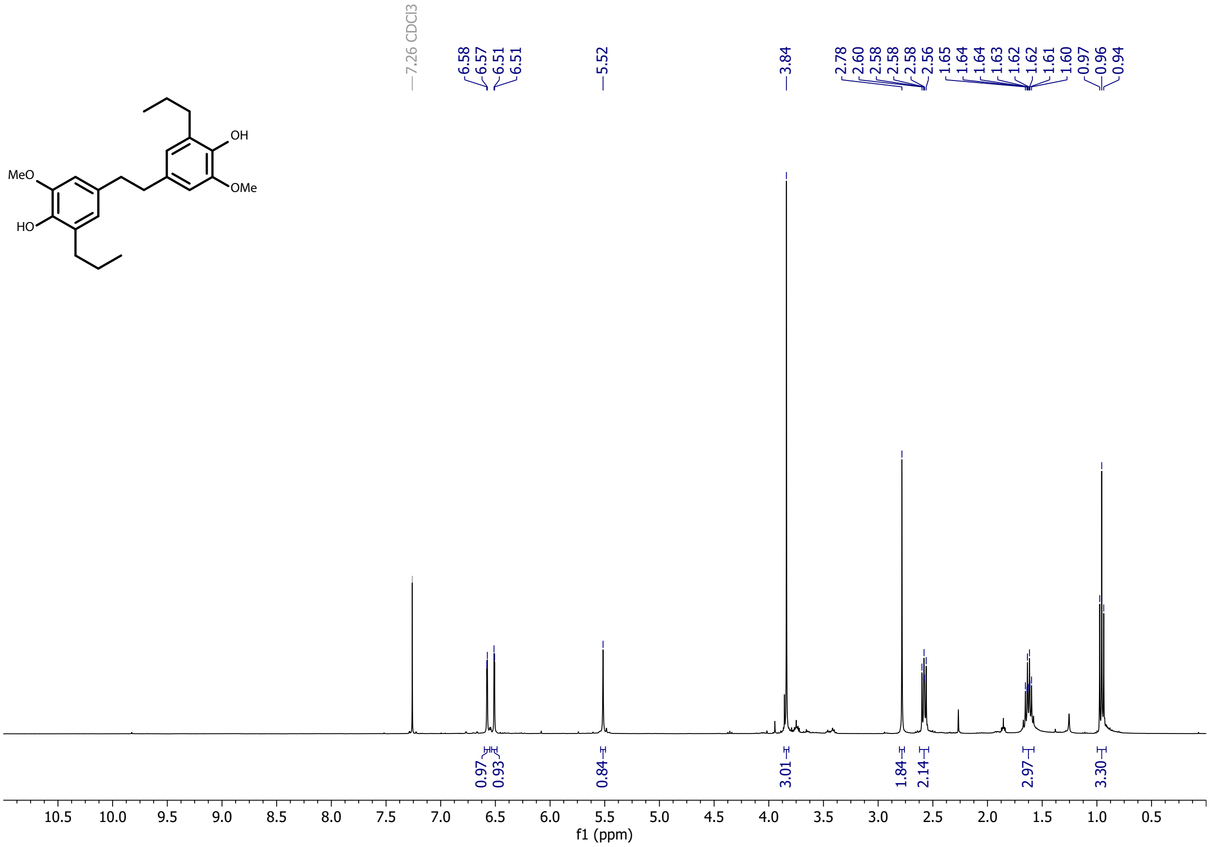

^{13}C NMR Spectrum (100 MHz, $CDCl_3$)

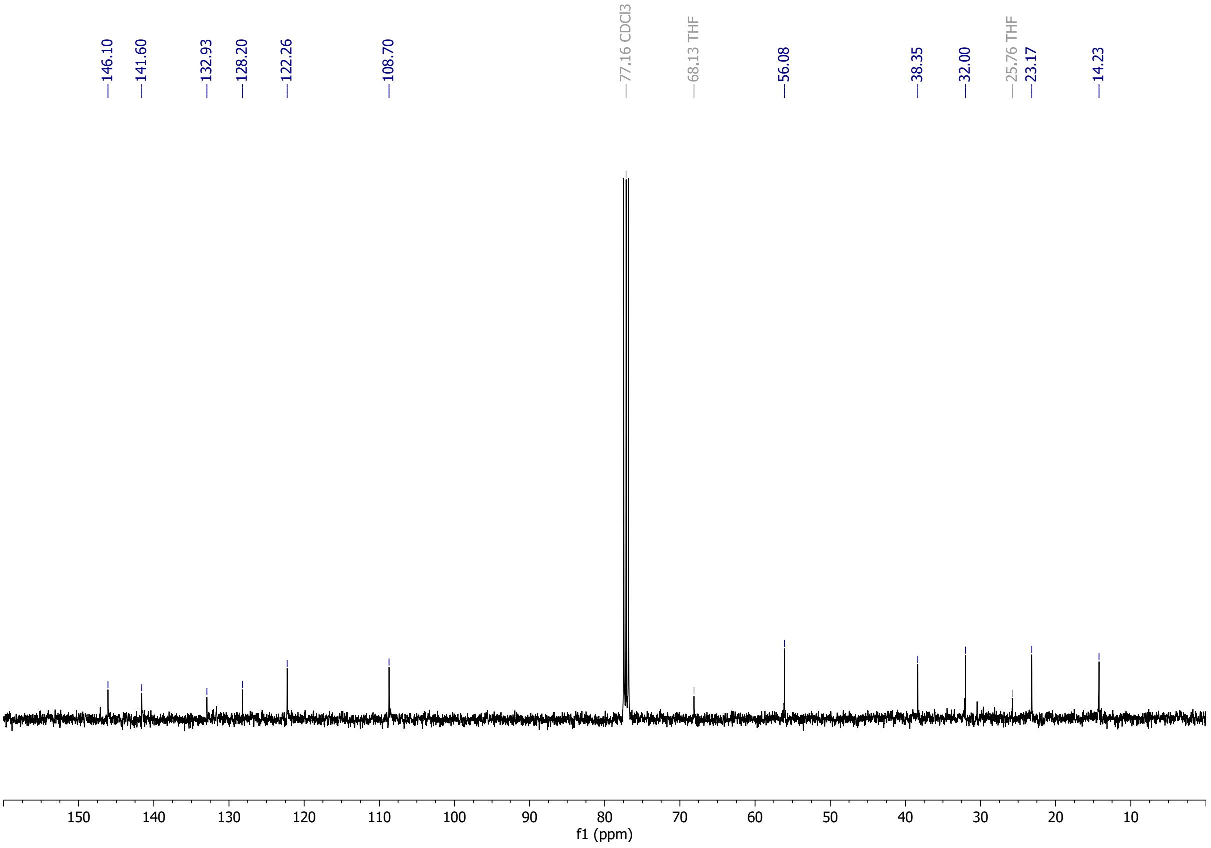

1,2-Bis(3,4-dimethoxy-5-propylphenyl)ethane (5d)

^{1}H NMR Spectrum (400 MHz, $CDCl_3$)

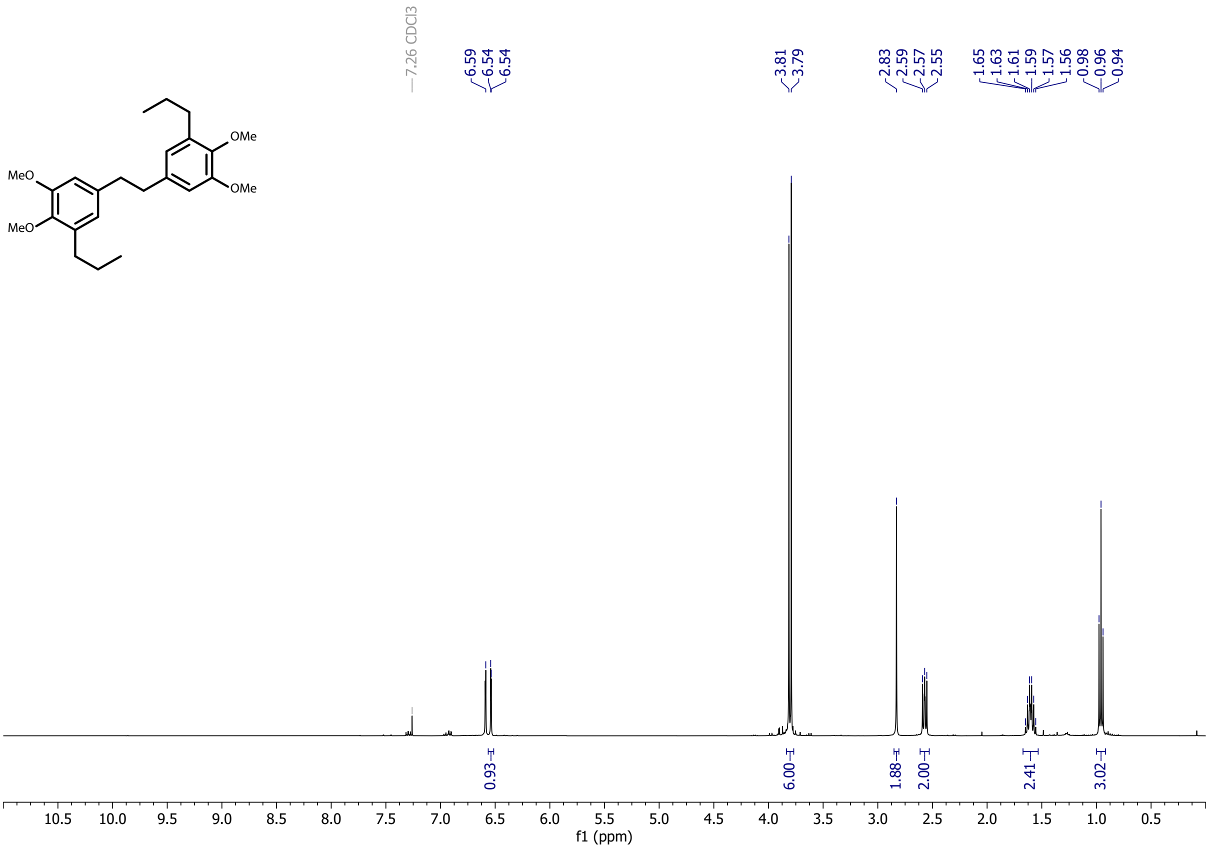

^{13}C NMR Spectrum (100 MHz, $CDCl_3$)

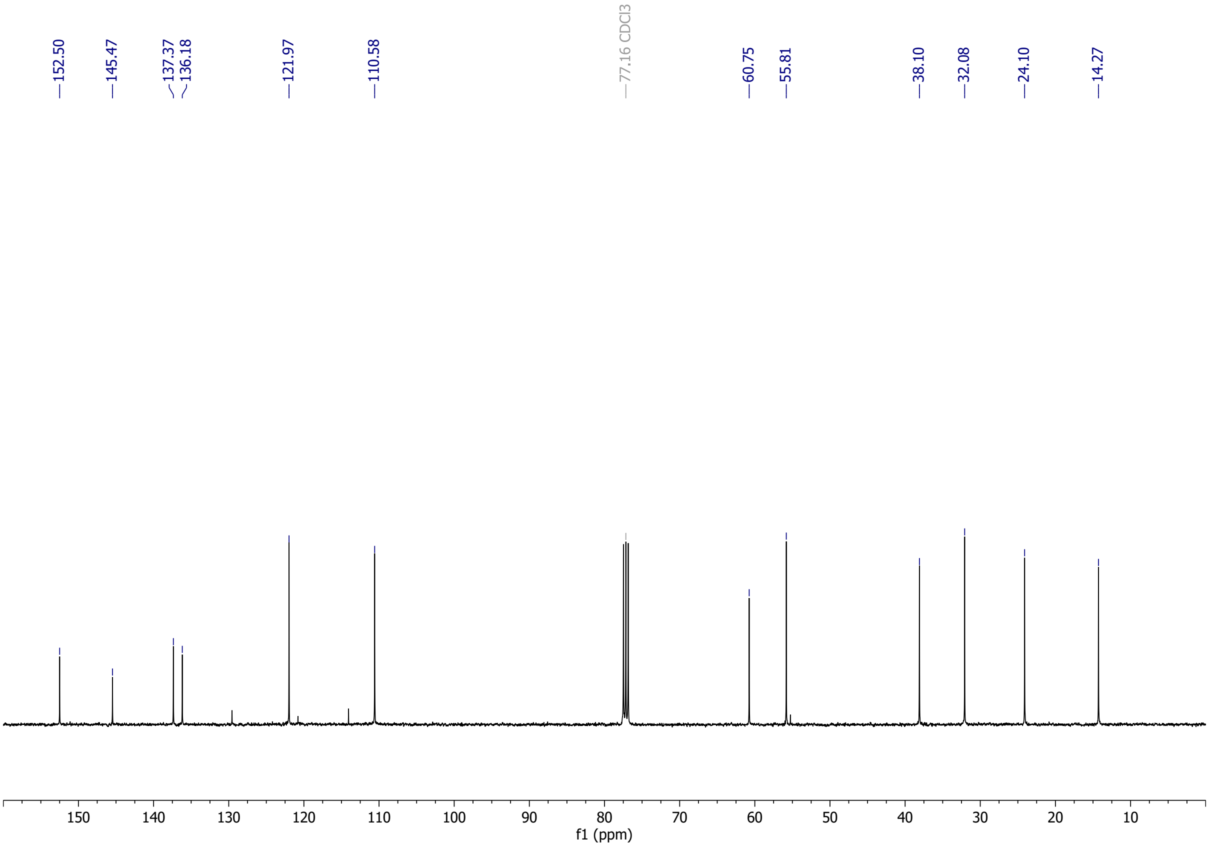

2,3,6,7-Tetramethoxy-4,5-dipropyl-9,10-dihydrophenanthrene (6d)

^{1}H NMR Spectrum (400 MHz, $CDCl_3$)

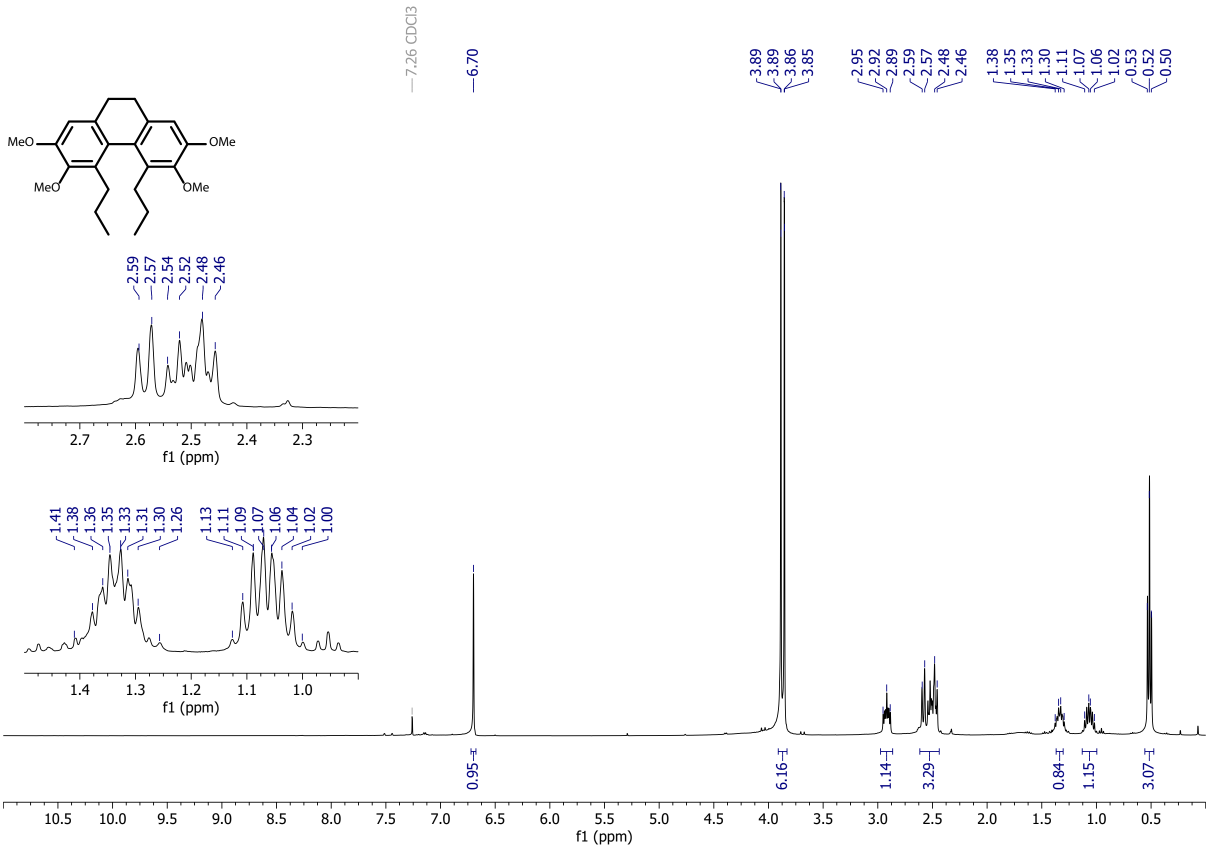

^{13}C NMR Spectrum (100 MHz, $CDCl_3$)

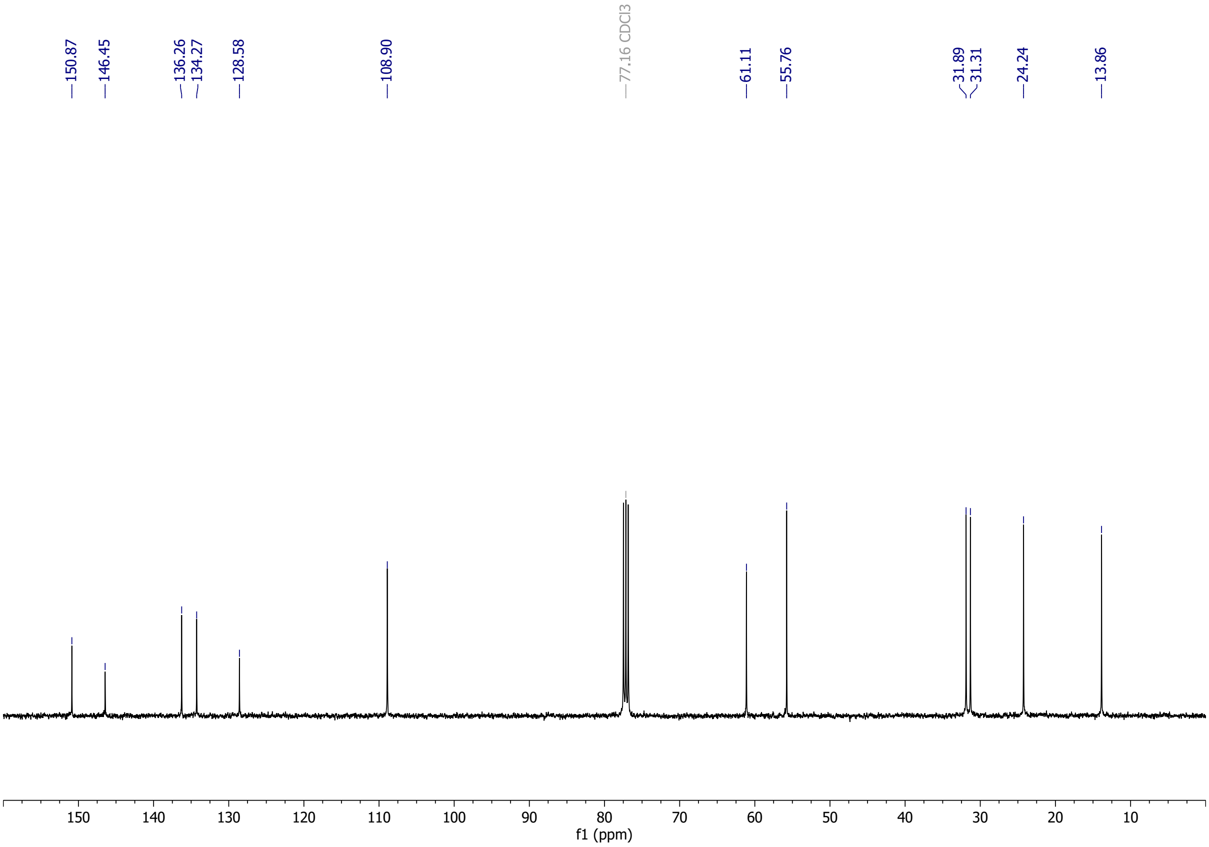

4,5-Dipropyl-9,10-dihydrophenanthrene-2,3,6,7-tetraol (1d)

^{1}H NMR Spectrum (400 MHz, CD_3OD)

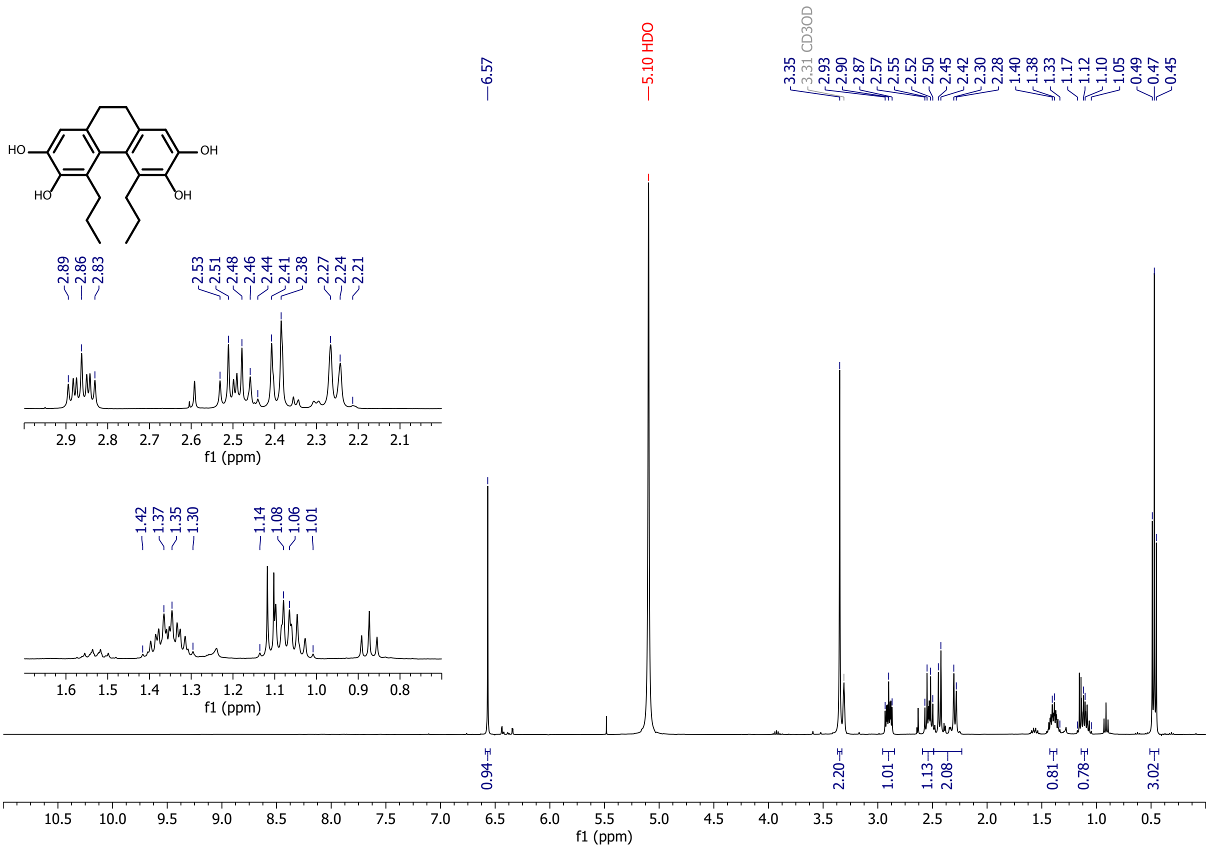

^{13}C NMR Spectrum (100 MHz, CD_3OD)

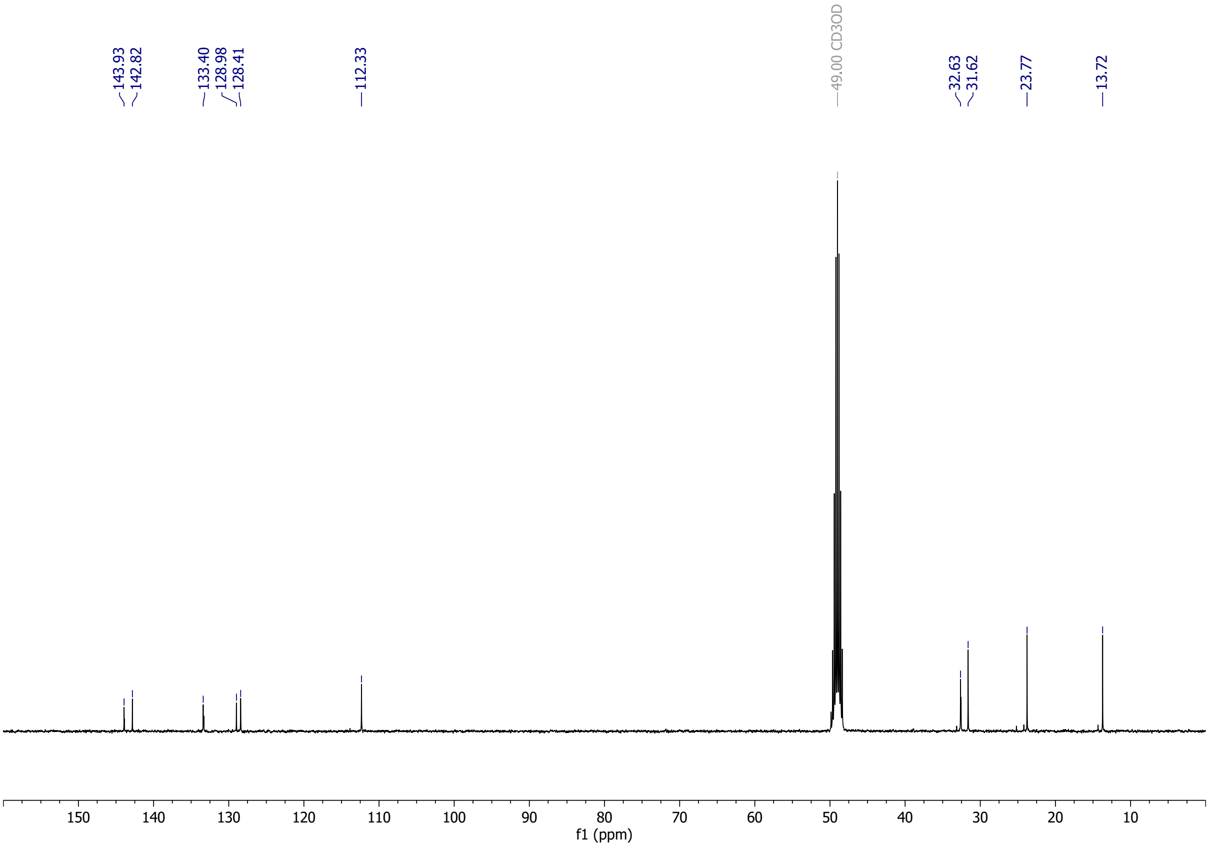

Comparison of ^{1}H NMR resonances for the dimethylene bridges of 4,5-disubstituted 9,10-phenanthrenes 6b-d and 1b-d in the region 3.00 – 2.00 ppm

6b-d ($CDCl_3$)

6b (Chloro)

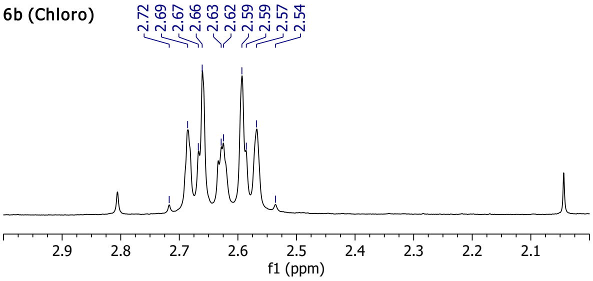

6c (Fluoro)

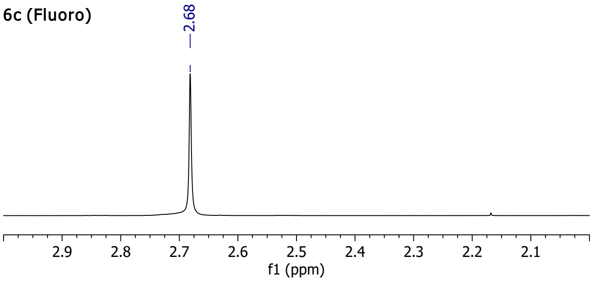

6d (Propyl)

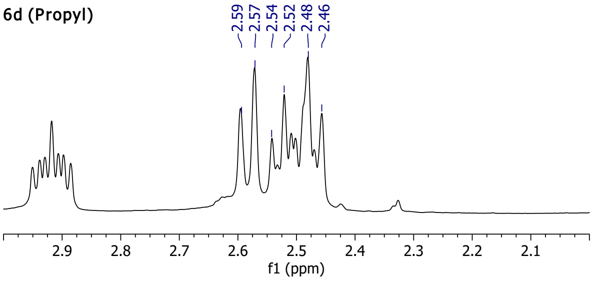

1b-d (CD_3OD)

1b (Chloro)

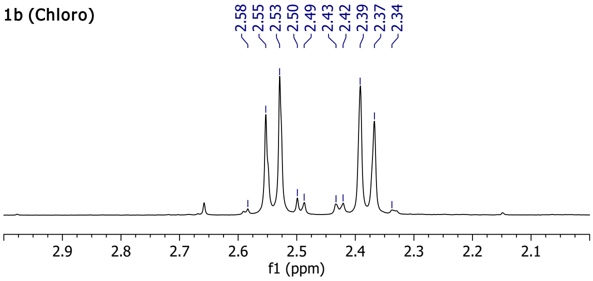

1c (Fluoro)

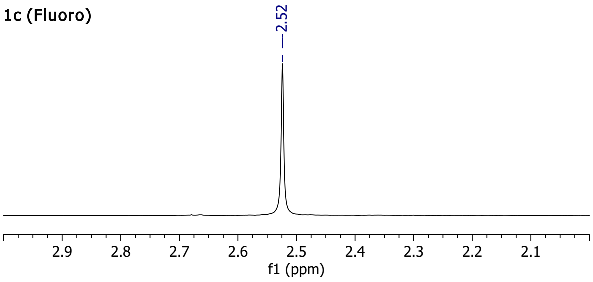

1d (Propyl)

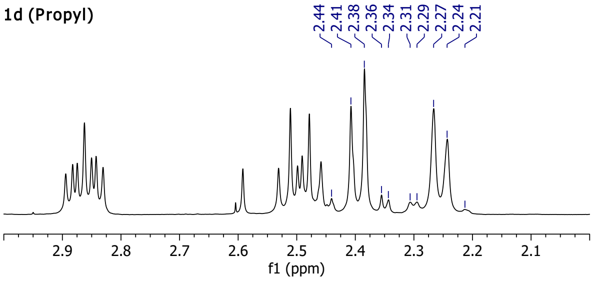

Analysis of the AA'XX' 'half-spectrum' multiplet at δ 153.4 ppm for the fluorine-bearing carbons in the ^{13}C NMR spectrum of 6c

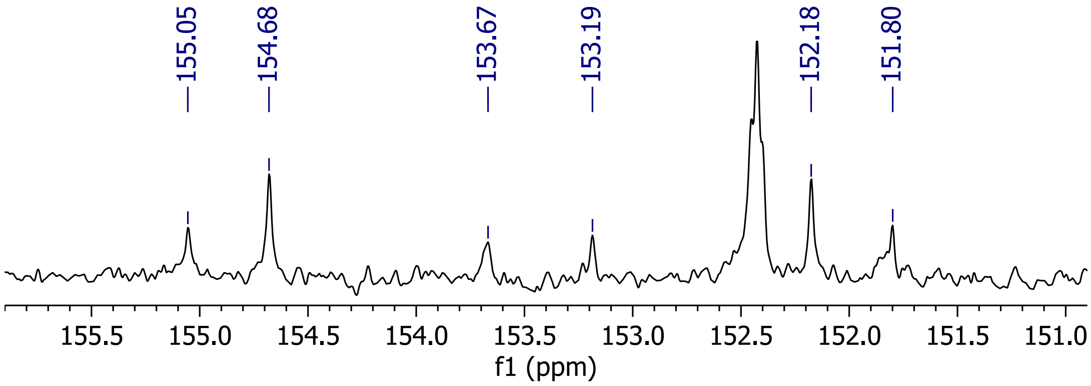

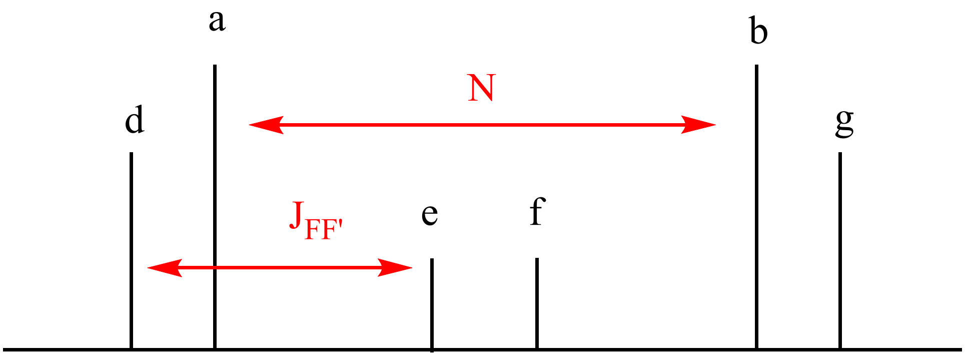

According to the method of Günther:[1]

$$N = J_{FC} + J_{FC'} = a - b$$

$$L = J_{FC} - J_{FC'} = \sqrt{(d-g)(e-f)}$$

$$J_{FF'} = d - e = f - g$$

Therefore:

$$J_{FC} = (N + L)/2$$

$$J_{FC'} = (N - L)/2$$

So:

$$N = 250\ Hz, L = 125\ Hz$$

$$J_{FF'} = 138\ Hz, J_{FC} = 188\ Hz, J_{FC'} = 62\ Hz$$

These calculated coupling constants are in an agreement with the values that are derived from the ^{13}C induced satellites in the ^{19}F spectrum, for which the values are:

$$J_{FC} = 188\ Hz, J_{FC'} = 63\ Hz$$

Table S1. Crystal Data, Data Collection and Refinement Parameters for the structures of **4b**, **5c**, **6a-d** and **7c**.

**data**	**4b**	**5c**	**6a**	**6b**
**formula**	$C_{16}H_{16}Cl_2O_4$	$C_{18}H_{20}F_2O_4$	$C_{18}H_{18}Br_2O_4$	$C_{18}H_{18}Cl_2O_4$
**formula weight**	343.19	338.34	458.14	369.22
**colour, habit**	colourless plates	colourless plates	colourless platy needles	colourless blocky needles
**temperature / K**	173	173	173	173
**crystal system**	monoclinic	monoclinic	monoclinic	orthorhombic
**space group**	*P*2/*c* (no. 13)	$P2_1/c$ (no. 14)	$P2_1/n$ (no. 14)	*Pbcn* (no. 60)
a **/ Å**	11.8634(5)	5.17807(20)	11.1133(6)	14.0686(8)
b **/ Å**	5.2240(2)	23.2653(11)	9.9384(4)	13.0445(6)
c **/ Å**	12.9213(6)	6.8867(3)	16.6772(7)	9.2392(5)
**α / deg**	90	90	90	90
**β / deg**	95.765(5)	102.070(4)	105.467(5)	90
**γ / deg**	90	90	90	90
V **/ Å**^{3}	796.73(7)	811.30(6)	1775.25(15)	1695.56(15)
Z	2 [c]	2 [c]	4	4 [d]
D_c **/ g cm**$^{-3}$	1.431	1.385	1.714	1.446
**radiation used**	Mo-Kα	Cu-Kα	Mo-Kα	Mo-Kα
**μ / mm**$^{-1}$	0.422	0.947	4.585	0.402
**2θ max / deg**	57	146	56	56
**no. of unique reflns**				
**measured (**R_{int}**)**	1613 (0.0180)	1562 (0.0267)	3554 (0.0301)	1728 (0.0182)
**obs,** $\|F_o\| > 4\sigma(\|F_o\|)$	1274	1100	2669	1409
**no. of variables**	105	111	221	112
R_1**(obs),** wR_2**(all)** [a]	0.0437, 0.1193	0.0427, 0.1264	0.0384, 0.0815	0.0343, 0.0793

Table S1. …continued.

data	6c	6d	7c
**formula**	$C_{18}H_{18}F_2O_4$	$C_{24}H_{32}O_4$	$C_{18}H_{16}F_2O_4$
**formula weight**	336.32	384.49	334.31
**colour, habit**	colourless blocky needles	colourless blocky needles	colourless blocky needles
**temperature / K**	173	173	173
**crystal system**	orthorhombic	orthorhombic	monoclinic
**space group**	$P2_12_12_1$ (no. 19)	*Pbca* (no. 61)	C2/*c* (no. 15)
a **/ Å**	9.0687(5)	8.3910(4)	25.183(2)
b **/ Å**	14.7626(6)	18.1238(12)	8.1656(8)
c **/ Å**	23.2602(9)	27.7790(12)	7.2689(7)
**α / deg**	90	90	90
**β / deg**	90	90	97.896(8)
**γ / deg**	90	90	90
V / Å^3	3114.0(2)	4224.6(4)	1480.6(2)
Z	8 [e]	8	4 [d]
D_c / g cm^{-3}	1.435	1.209	1.500
**radiation used**	Mo-Kα	Mo-Kα	Mo-Kα
**μ / mm^{-1}**	0.116	0.081	0.122
**2θ max / deg**	56	56	56
**no. of unique reflns**			
**measured (R_{int})**	5162 (0.0272)	4220 (0.0244)	1566 (0.0395)
**obs, $\|F_o\| > 4\sigma(\|F_o\|)$**	4340	3186	1259
**no. of variables**	442	260	111
R_1(obs), wR_2(all) [a]	0.0422, 0.0879	0.0474, 0.1030	0.0443, 0.1183

[a] $R_1 = \Sigma||F_o| - |F_c||/\Sigma|F_o|$; $wR_2 = \{\Sigma[w(F_o^2 - F_c^2)^2] / \Sigma[w(F_o^2)^2]\}^{1/2}$; $w^{-1} = \sigma^2(F_o^2) + (aP)^2 + bP$. [c] The molecule has crystallographic C_i symmetry. [d] The molecule has crystallographic C_2 symmetry. [e] There are two crystallographically independent molecules.

Table S1 provides a summary of the crystallographic data for the structures of **4b**, **5c**, **6a**, **6b**, **6c**, **6d** and **7c**. Data were collected using Agilent Xcalibur 3 E (of **4b**, **6a**, **6b**, **6c**, **6d** and **7c**) and Xcalibur PX Ultra A (**5c**) diffractometers, and the structures were refined using the SHELXTL and SHELX-2013 program systems.[2,3] The absolute structure of **6c** could not be determined [Flack parameter $x = 0.5(7)$, see below]. CCDC 1947920 to 1947926.

X-ray crystallography

The structure of **4b** was found to sit across a centre of symmetry at the middle of the C7–C7A bond. The unique O8–H hydrogen atom was located from a ΔF map and refined freely subject to an O–H distance constraint of 0.90 Å.

The structure of **5c** was found to sit across a centre of symmetry at the middle of the C1–C1A bond.

The structure of **6b** was found to sit across a C_2 axis that bisects the C1–C1A and C7–C7A bonds.

The structure of **6c** was found to crystallise in the chiral space group $P2_12_12_1$ with two independent molecules, **6c-A** and **6c-B**, in the asymmetric unit. The absolute structure could not be determined, with the Flack parameter from the final refinement being 0.5(7). This is not surprising as the two independent molecules are, with the exception of the C16 methyl group, enantiomers of each other, the r.m.s. fit of molecule A with inverted molecule B (less C16) being *ca*. 0.13 Å. The structure can, in fact, be solved and refined in the centrosymmetric space group *Pbca* with one independent molecule in the asymmetric unit, but in addition to the disorder of C16, the thermal ellipsoids of a number of the atoms behave poorly with an *R*-factor of *ca*. 15%. Overall, this is a much lower quality model of the structure, and so the two independent molecules in $P2_12_12_1$ approach has been preferred.

The structure of **7c** was found to sit across a C_2 axis that bisects the C1–C1A and C7–C7A bonds.

Figures

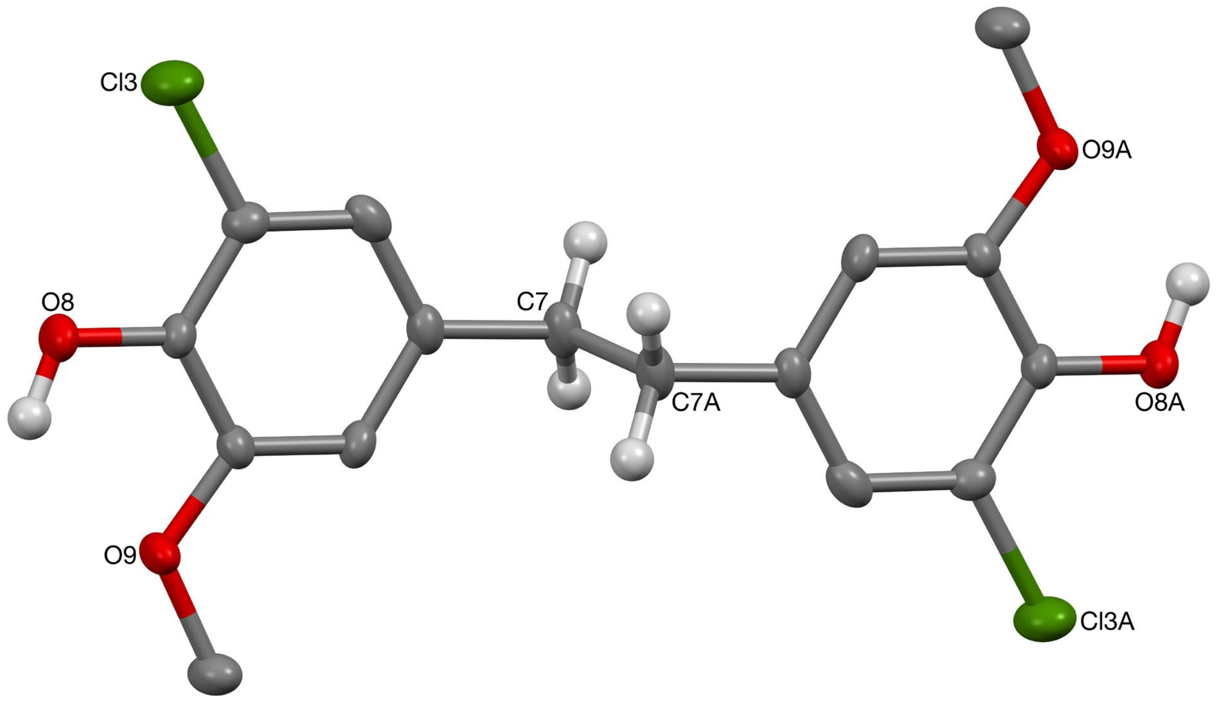

Fig. S1 The crystal structure of **4b** (50% probability ellipsoids). The molecule sits across a centre of symmetry at the middle of the C7–C7A bond.

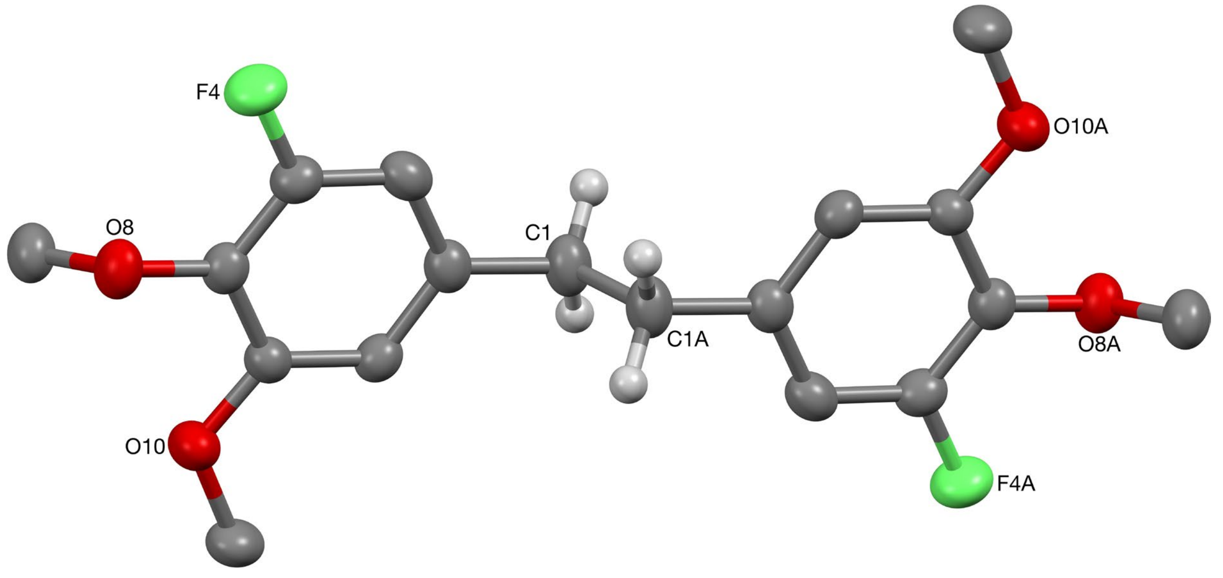

Fig. S2 The crystal structure of **5c** (50% probability ellipsoids). The molecule sits across a centre of symmetry at the middle of the C1–C1A bond.

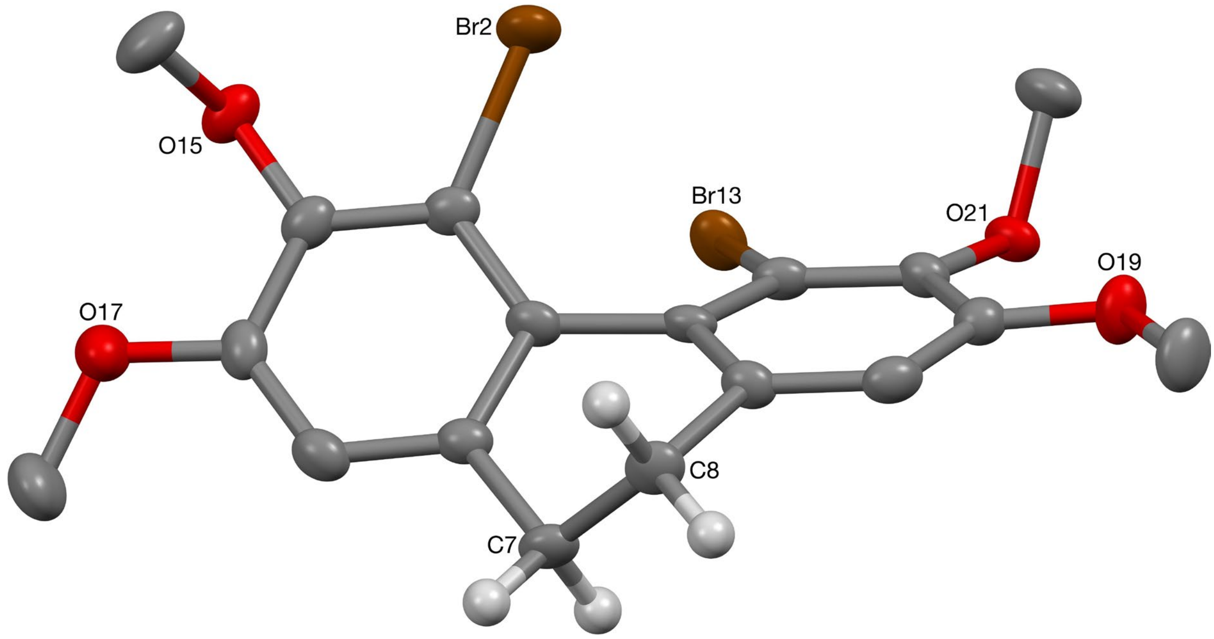

Fig. S3 The crystal structure of **6a** (50% probability ellipsoids).

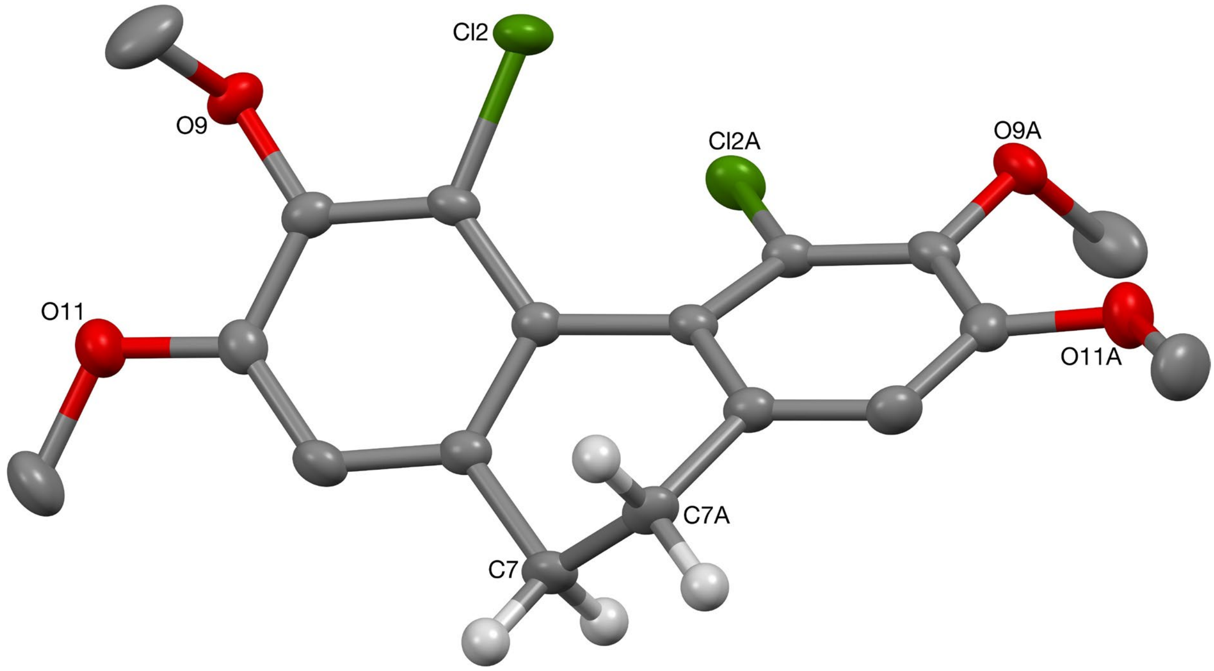

Fig. S4 The crystal structure of **6b** (50% probability ellipsoids). The molecule sits across a C_2 axis that bisects the C7–C7A bond and the Cl2···Cl2A vector.

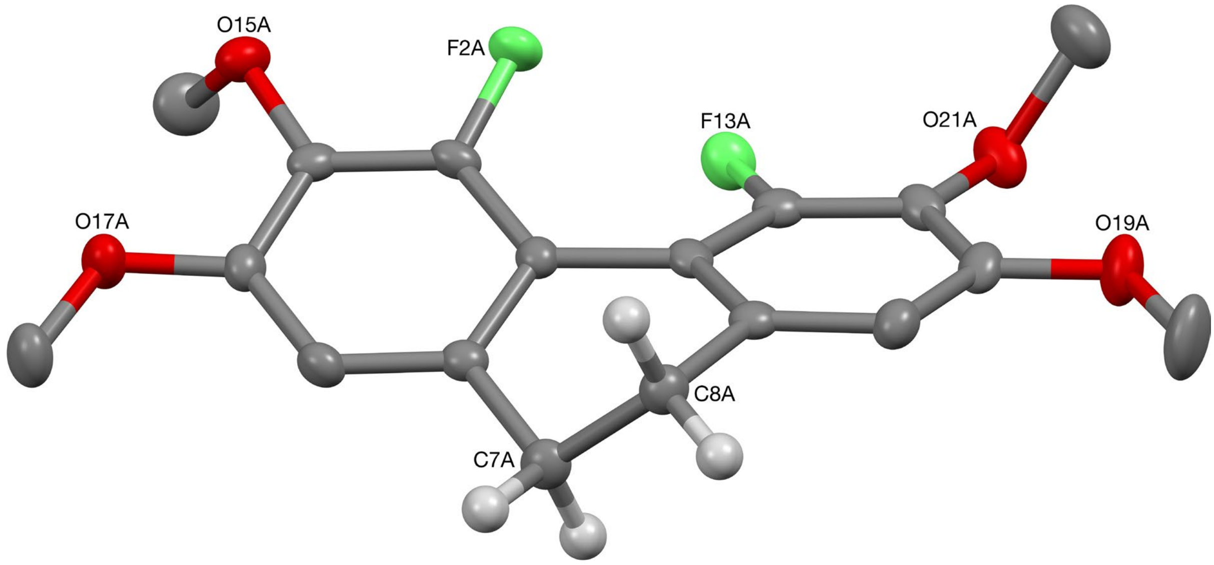

Fig. S5 The structure of **6c-A**, one of the two independent molecules present in the crystal of **6c** (50% probability ellipsoids).

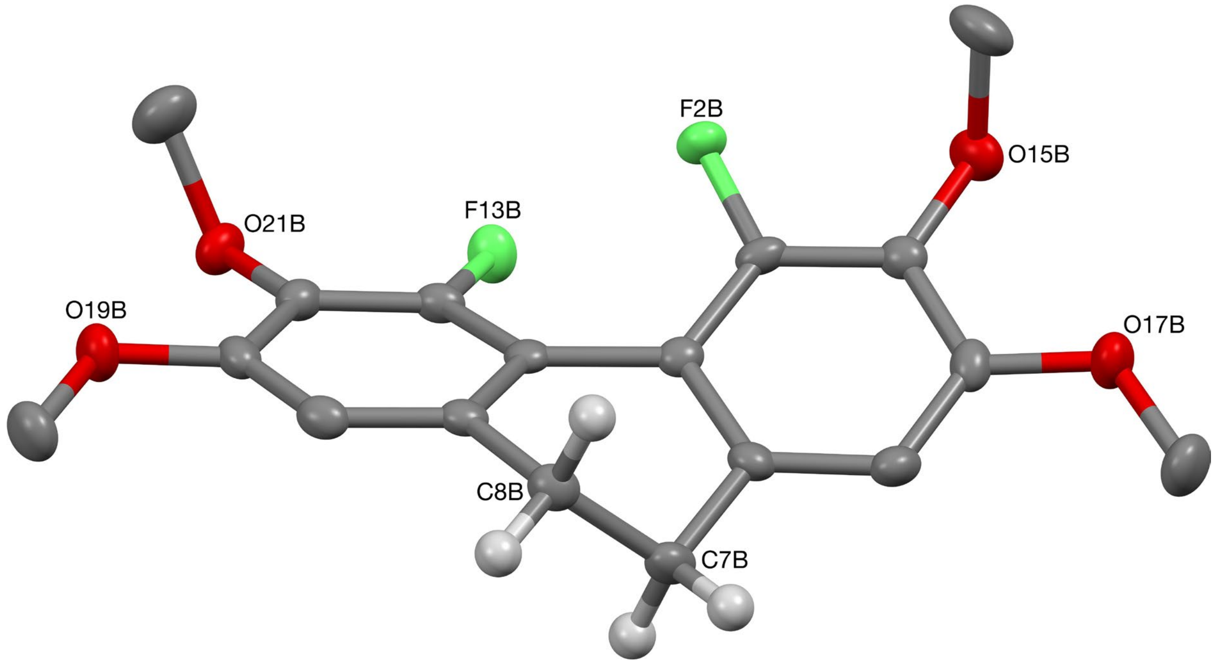

Fig. S6 The structure of **6c-B**, one of the two independent molecules present in the crystal of **6c** (50% probability ellipsoids).

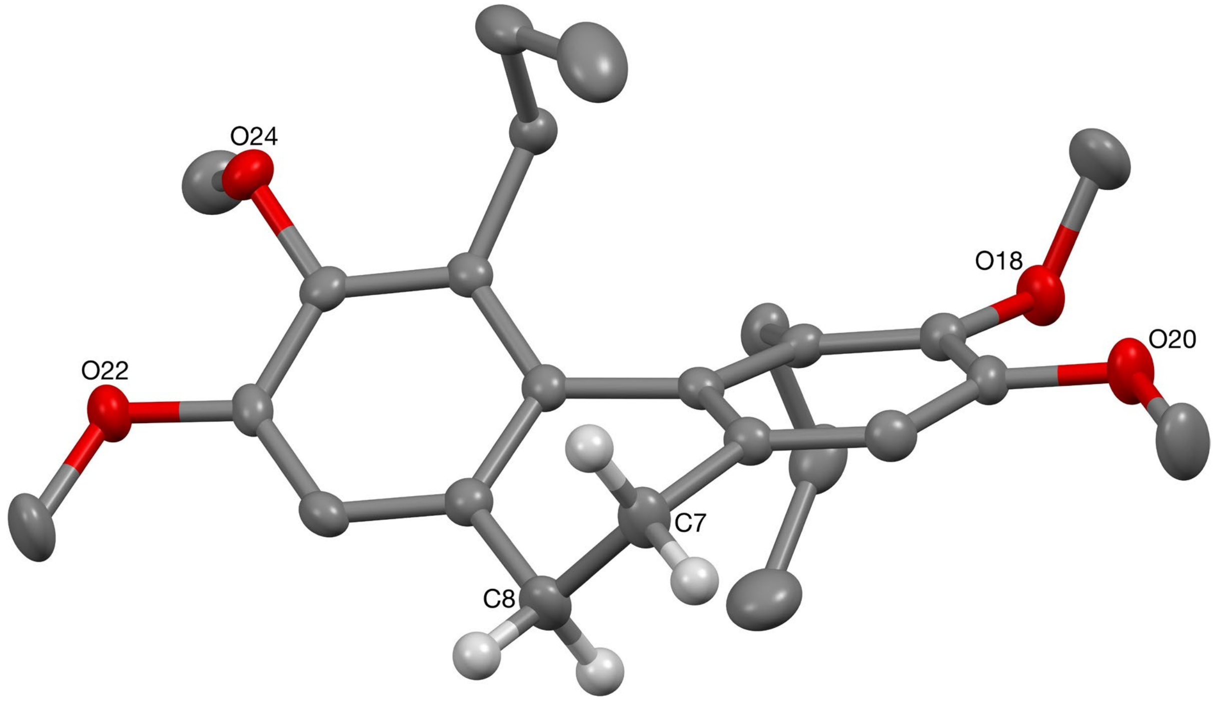

Fig. S7 The crystal structure of **6d** (50% probability ellipsoids).

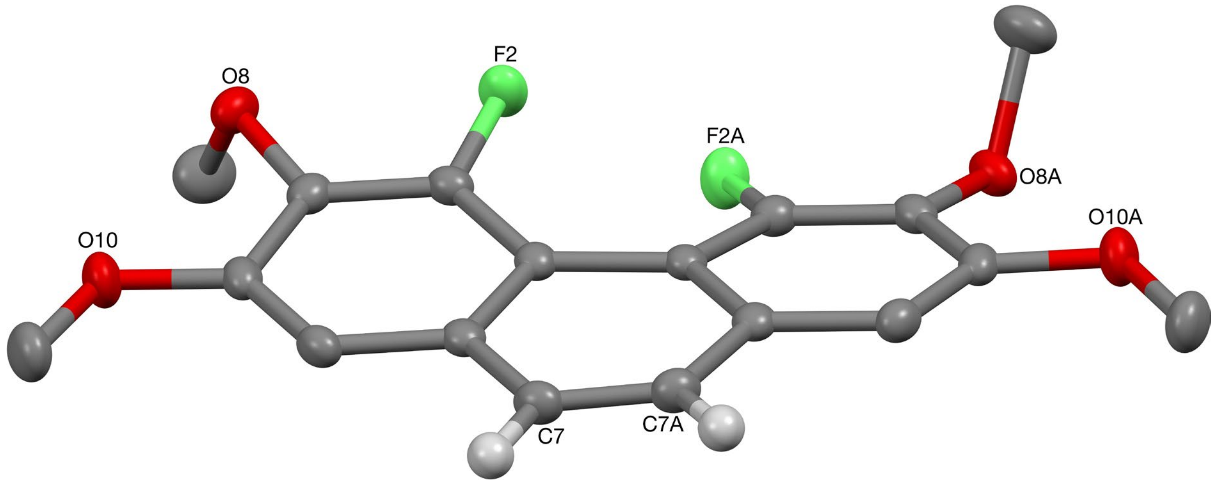

Fig. S8 The crystal structure of **7c** (50% probability ellipsoids). The molecule sits across a C_2 axis that bisects the C7–C7A bond and the F2···F2A vector.

CSP-HPLC chromatograms for 6a-d

4,5-Dibromo-2,3,6,7-tetramethoxy-9,10-dihydrophenanthrene (6a)

CHIRALPAK AD; 99% n-hexane, 1% isopropanol; 240 nm; t_R = 18.1 min, 25.3 min – 49.7:50.3 e.r.

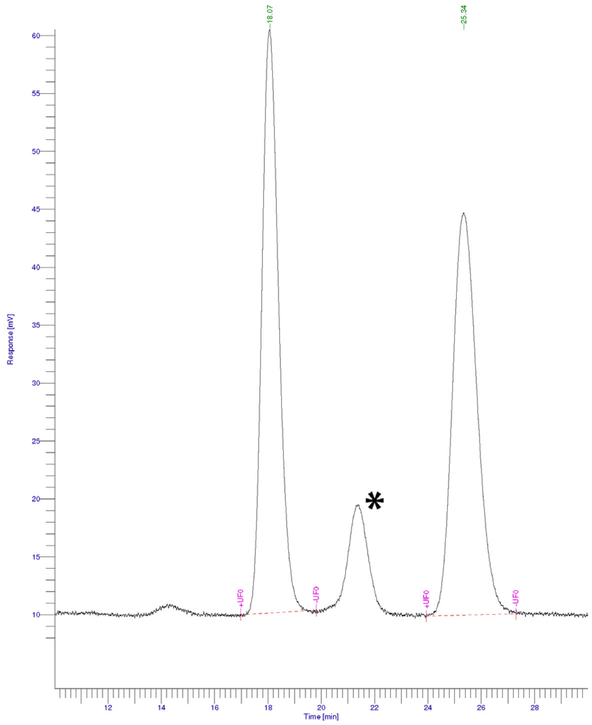

4,5-Dichloro-2,3,6,7-tetramethoxy-9,10-dihydrophenanthrene (6b)

CHIRALPAK AD; 99% n-hexane, 1% isopropanol; 240 nm; t_R = 15.0 min, 20.5 min – 49.6:50.4 e.r.

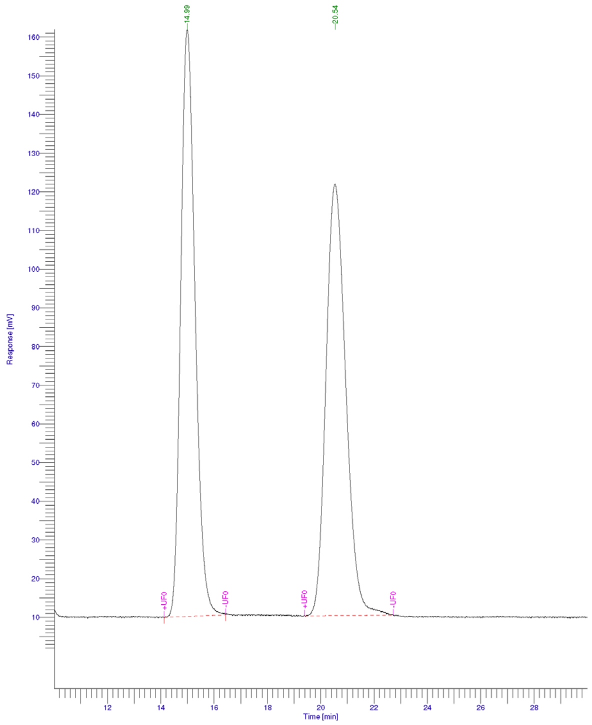

4,5-Difluoro-2,3,6,7-tetramethoxy-9,10-dihydrophenanthrene (6c)

CHIRALPAK AD; 99% n-hexane, 1% isopropanol; 240 nm; t_R = 18.0 min.

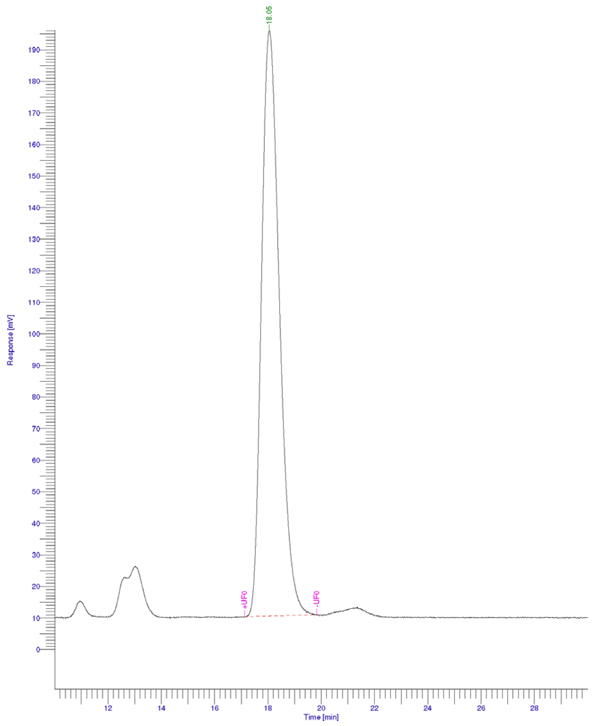

2,3,6,7-Tetramethoxy-4,5-dipropyl-9,10-dihydrophenanthrene (6d)

CHIRALPAK AD; 100% n-hexane; 240 nm; t_R = 5.7 min, 6.2 min – 49.5:50.5 e.r.

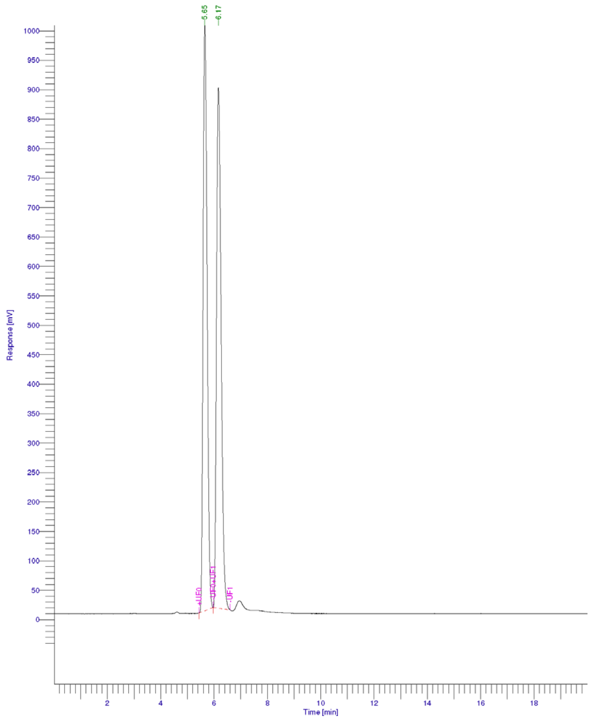

*** 4,5-Dibromo-2,3,6,7-tetramethoxyphenanthrene impurity**

References

1. H. Günther, *Angew. Chem. Int. Ed.*, 1972, **11**, 861-874.

2. SHELXTL v5.1, Bruker AXS, Madison, WI, 1998.SHELXTL v5.1, Bruker AXS, Madison, WI, 1998.

3. SHELX-2013, G.M. SHELX-2013, G.M. Sheldrick, *Acta Cryst.*, 2015, **C71**, 3-8.
